# Inequalities in early marriage, childbearing and sexual debut among adolescents in sub-Saharan Africa

**DOI:** 10.1186/s12978-021-01125-8

**Published:** 2021-06-17

**Authors:** Dessalegn Y. Melesse, Réka M. Cane, Aveneni Mangombe, Macellina Y. Ijadunola, Adom Manu, Eniola Bamgboye, Abdu Mohiddin, Rornald M. Kananura, Elsie Akwara, Elsabé du Plessis, Yohannes D. Wado, Martin K. Mutua, Wubegzier Mekonnen, Cheikh M. Faye, Sarah Neal, Ties Boerma

**Affiliations:** 1 Countdown To 2030 for Women’s, Children’s and Adolescents’ Health, Institute for Global Public Health, University of Manitoba, R070 Med Rehab Building, 771 McDermot Avenue, Winnipeg, MB R3E 0T6 Canada; 2 Women’s and Children’s Health Program, Instituto Nacional de Saúde, Ministério da Saúde, Maputo, Mozambique; 3 Ministry of Health and Child Care, Harare, Zimbabwe; 4 Obafemi Awolowo University, Ile-Ife, Nigeria; 5 University of Ghana, Accra, Ghana; 6 Department of Epidemiology and Medical Statistics, University of Ibadan, Ibadan, Nigeria; 7 Centre of Excellence in Women and Child Health, Aga Khan University, Nairobi, Kenya; 8 Department of Health Planning and Management, Makerere University School of Public Health, Kampala, Uganda; 9 World Health Organization, Geneva, Switzerland; 10 African Population and Health Research Center, Nairobi, Kenya; 11 School of Public Health, Addis Ababa University, Addis Ababa, Ethiopia; 12 African Population and Health Research Center, Dakar, Senegal; 13 Department of Social Statistics and Demography, University of Southampton, Hampshire, UK

**Keywords:** Adolescents, Girls, Boys, Child marriage, Childbearing, Sexual debut, Sub-Saharan Africa, Inequalities, Trends and patterns, Geographical disparities

## Abstract

**Background:**

Adolescent sexual and reproductive health (ASRH) is a major public health concern in sub-Saharan Africa (SSA). However, inequalities in ASRH have received less attention than many other public health priority areas, in part due to limited data. In this study, we examine inequalities in key ASRH indicators.

**Methods:**

We analyzed national household surveys from 37 countries in SSA, conducted during 1990–2018, to examine trends and inequalities in adolescent behaviors related to early marriage, childbearing and sexual debut among adolescents using data from respondents 15–24 years. Survival analyses were conducted on each survey to obtain estimates for the ASRH indicators. Multilevel linear regression modelling was used to obtain estimates for 2000 and 2015 in four subregions of SSA for all indicators, disaggregated by sex, age, household wealth, urban–rural residence and educational status (primary or less versus secondary or higher education).

**Results:**

In 2015, 28% of adolescent girls in SSA were married before age 18, declined at an average annual rate of 1.5% during 2000–2015, while 47% of girls gave birth before age 20, declining at 0.6% per year. Child marriage was rare for boys (2.5%). About 54% and 43% of girls and boys, respectively, had their sexual debut before 18. The declines were greater for the indicators of early adolescence (10–14 years). Large differences in marriage and childbearing were observed between adolescent girls from rural versus urban areas and the poorest versus richest households, with much greater inequalities observed in West and Central Africa where the prevalence was highest. The urban–rural and wealth-related inequalities remained stagnant or widened during 2000–2015, as the decline was relatively slower among rural and the poorest compared to urban and the richest girls. The prevalence of the ASRH indicators did not decline or increase in either education categories.

**Conclusion:**

Early marriage, childbearing and sexual debut declined in SSA but the 2015 levels were still high, especially in Central and West Africa, and inequalities persisted or became larger. In particular, rural, less educated and poorest adolescent girls continued to face higher ASRH risks and vulnerabilities. Greater attention to disparities in ASRH is needed for better targeting of interventions and monitoring of progress.

**Supplementary Information:**

The online version contains supplementary material available at 10.1186/s12978-021-01125-8.

## Background

The global adolescent population (10–19) is estimated to have reached 1.3 billion (49%: 15–19 years old) in 2020, of whom over 235 million (46%: 15–19 years old) live in sub-Saharan Africa (SSA), accounting for 23% of the region’s population [1]. Healthy adolescence is critical for the achievement of the Sustainable Development Goals (SDGs), including those related to health, education, poverty, security, and reduction of inequalities [2, 3], in particular adolescent sexual and reproductive health (ASRH).

ASRH risk behaviours are associated with unintended pregnancies, early sexual initiation (often coerced), early marriage, HIV and other sexually transmitted infections (STIs)[4, 5]. Early marriage disproportionately affects girls, while unintended pregnancy often leads to school dropout and compromises educational advancement and human capital development [6–8]. Early childbearing is also associated with increased health risks for mothers and newborns [9]. Furthermore, premarital and extramarital sex carry risks of unintended pregnancy, which may lead to unsafe abortion, and contracting STIs including HIV. Many of these risks are greatest among younger adolescents, yet most analyses fail to recognise and report events for this age group [9–12].

Inequalities in sexual and reproductive health persist as existing reviews document higher rates of early marriage and childbearing among adolescents who are poorer, less educated and from rural areas [13, 14]. Recent evidence shows that early marriage and childbearing among adolescents in SSA has declined, most notably among urban and better-educated populations [13–16]. Age at sexual debut among adolescents in SSA has been increasing, with some studies showing a relatively lower proportion of boys and girls reportedly initiating sexual intercourse before age 15 [17–19].

A better understanding of ASRH challenges in SSA, based on disaggregated data for critical adolescent transitions, is crucial to strengthen policies and programs targeting adolescent needs^14^,^ 16^,^ 20^. Much of our current knowledge is based on analyses of aggregate data and on single-topics. However, the key life events during adolescence, such as marriage, childbearing and sexual debut, are intertwined and need to be systematically synthesized as such for greater understanding of ASRH-related issues. The lack of disaggregated data about adolescents implies that their specific needs and vulnerabilities remain largely invisible to policy and program designers, and present challenges to achieving the SDG equity agenda.

This paper examines three dimensions of inequalities in ASRH indicators, child marriage, childbearing and sexual debut, in SSA as a whole and in four geographic subregions during 2000–2015 using national surveys conducted between 1990 and 2018. We use the World Health Organization (WHO) definition of adolescents as aged between 10 and 19 years and those between 10–14 and 15–19 years of age hereafter are referred as younger and older adolescents, respectively [21].

## Methods

The data used in this study were drawn from nationally representative Demographic and Health Surveys (DHS) and AIDS Indicator Surveys, which collect health and socio-demographic information using the same survey methodology [22]. Data for adolescents and young people (15–24 years at time of survey) were extracted from 129 national surveys conducted since 1990 in 37 countries in SSA, of which 32 countries had conducted at least two surveys and 33 at least one survey during 2010–2018 (Additional file 1: Appendix Table S1).

The key indicators were first marriage or consensual union (or cohabitation as if married, hereafter simply called marriage) before age 18, often referred to as child marriage[23]; childbirth before age 20 which is related to the SDG indicator of adolescent birth rate[24]; and sexual debut before age 18. We also applied 15 years as an age cut-off for all three indicators to gain insight into the trends and inequalities during early adolescence (10–14 years).

We used data on reported current status on marriage, childbirth and sexual debut by age of the respondent (e.g., are you currently married) and recalled age at first event (e.g., age at first childbirth) from respondents 15–24 years to obtain a sufficiently large sample. To account for censoring we used Kaplan–Meier survival analysis in which the survival time to experiencing each event before a specified age was obtained from a cumulated single-year percent distribution from the product-limit estimates of the survival curve (sts function in the statistical package of Stata version 15[25]). We derived our indicator values from the survival curves. Child marriage and childbearing were measured by the cumulative probability of being married before the age of 18 years and having first birth before the age of 20 years, respectively. The distribution of timing of first sex was estimated in the same way as age at first marriage by age 18 years. A similar approach was used to explore the distribution of the three key life events occurring before age 15 years. For details see Additional file 1.

Gender-specific disaggregated analyses were conducted by urban–rural residence, education status and socioeconomic status for SSA. In this paper, we present the results for girls and boys but limited the disaggregated analysis to girls given the low prevalence of marriage among adolescent boys. Educational status was categorized into two groups as primary or less education and secondary or higher education, based on highest completed level of education at time of the interview. We computed and used wealth tertiles rather than the conventional wealth quintiles from the standard DHS dataset index scores [26] to reduce sampling errors.

Using the estimates obtained from survival analysis, multilevel mixed-effects linear regression analyses were performed on year of survey to derive crude estimates of average trends for the key indicators for SSA and subregions. The multilevel data used for analysis is characterized by a hierarchical or multilevel structure, where we have countries—each with multiple date points measured from 1990 to 2018—nested within the sub-regions and sub-regions nested within the bigger region of SSA. Informed by the United Nations Population Division subregional classification [27], countries were grouped into four subregions (Central, West, Eastern and Southern Africa) of SSA (Additional file 1: Appendix Table S1). In order to obtain better estimates for the period 2000–2015, a priori knowledge obtained from pre-2000 survey data points were used where available. The use of surveys prior to 2000 provided a more accurate estimate to measure the trends compared to using only surveys after 2000 or two end data points on year 2000 and 2015. We computed confidence intervals for all estimates in 2000 and 2015 where relevant, tested for statistical significance of changes over time using a standard approach [28] and used the regression coefficient estimates to obtain the average annual rate of change (AARC). These estimates of confidence intervals and p-values are available for reference in respective tables. Further details are provided in the Additional file 1.

## Results

### General levels and trends

**Fig. 1:**
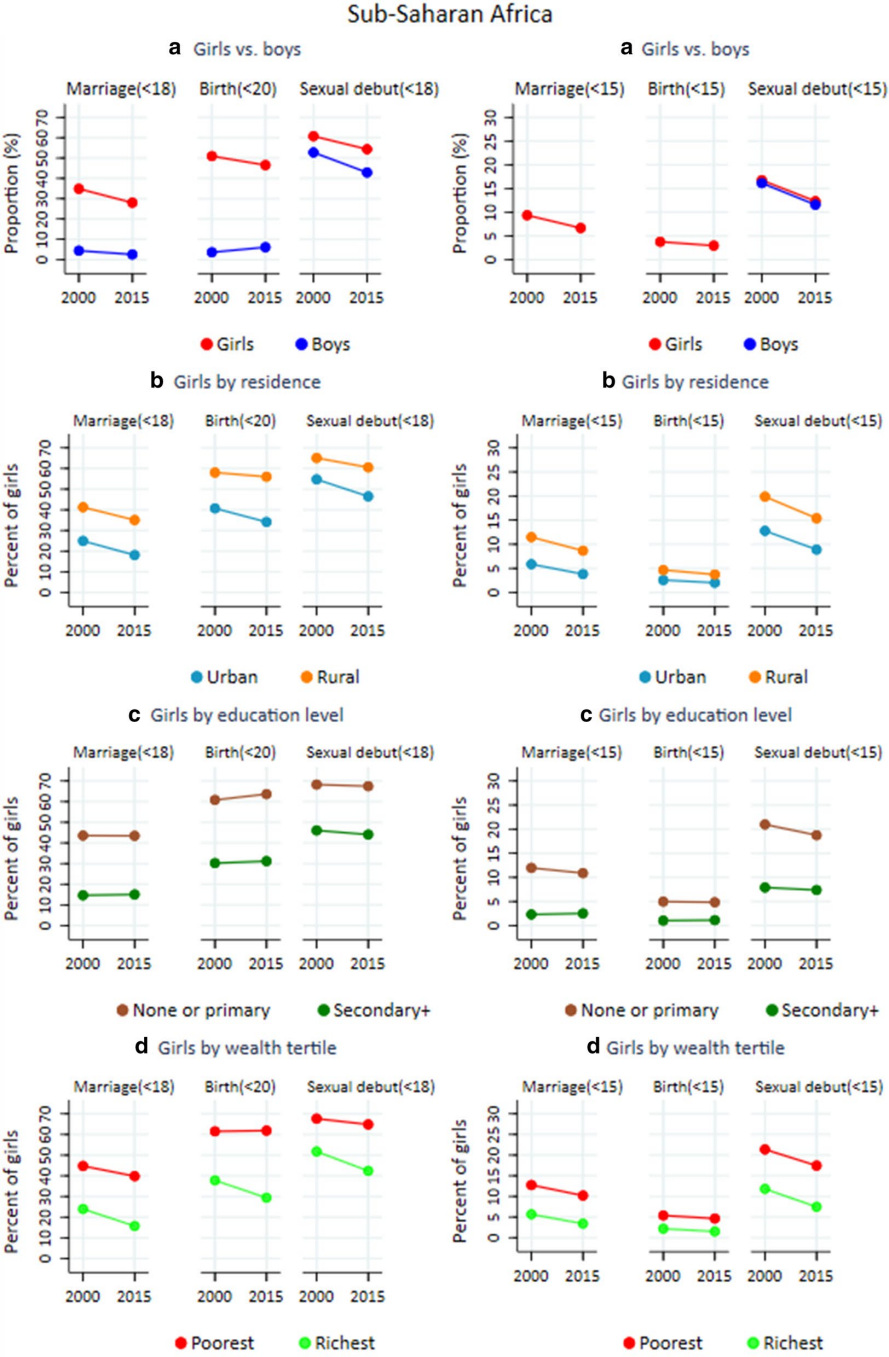
**a**–**d** Percentages of child marriage (age 18), childbearing (age 20) and sexual debut (age 18) among adolescents in sub-Saharan Africa, disaggregated by sex, and by residence and socioeconomic characteristics for girls, based on national surveys in 37 countries conducted during 1990–2018, 37 countries, sub-Saharan Africa

In 2015, 28% of adolescent girls in SSA were married before age 18, declining from 35% in 2000 at an average annual rate of 1.4% (Table 1 and Fig. 1a). Fewer boys (2.5%) were married by age 18 in 2015, a decline from 4.4% in 2000 at an average annual rate of 3.6%. Childbearing before age 20 declined at a rate of 0.6% per year in recent decades to reach 46.5% in 2015. Sexual activity before age 18 in SSA decreased from 61 to 54% for girls and from 53 to 43% for boys during 2000–2015 (AARC: − 0.7% and − 1.3% respectively).

**Table 1:** Levels and trends of child marriage, childbearing and sexual debut among adolescents by sex and sub-region in Sub-Saharan Africa, based on national surveys 1990–2018 in 37 countries, sub-Saharan Africa, in 2000 and 2015 (in parenthesis 95% confidence intervals)

	Before age 18 (20 for childbearing)				Before age 15			
	2000	2015	AARC	p	2000	2015	AARC	p
Sub-Saharan Africa								
Marriage								
F	34.8 [25.5, 44.2]	28.0 [18.6, 37.4]	− 1.4	0.32	9.4 [5.9, 12.9]	6.7 [3.1, 10.2]	− 2.3	0.29
M	4.4 [3.4, 5.3]	2.5 [1.0, 4.1]	− 3.6	0.04	***	***	***	
Childbearing								
F	50.9 [47.0, 54.8]	46.5 [42.5, 50.5]	− 0.6	0.12	3.8 [2.5, 5.0]	2.9[1.7, 4.2]	− 1.6	0.38
Sexual debut								
F	60.7 [51.7, 69.7]	54.3 [45.2, 63.3]	− 0.7	0.33	16.7 [11.8, 21.7]	12.3 [7.3, 17.3]	− 2.0	0.22
M	52.7 [43.5, 61.9]	42.8 [33.5, 52.1]	− 1.3	< 0.001	16.1 [12.0, 20.3]	11.6 [7.4, 15.8]	− 2.2	0.13
Central Africa								
Marriage								
F	42.6 [34.8, 50.3]	32.4 [24.4, 40.4]	− 1.8	0.07	12.0 [7.9, 16.2]	8.6 [4.3, 12.9]	− 2.2	0.27
M	5.1 [3.5, 6.7]	3.9 [0.8, 7.0]	− 1.7	0.51	***	***	***	
Childbearing								
F	57.8 [52.9, 62.6]	51.4 [46.0, 56.7]	− 0.7	0.08	4.8 [3.3, 6.4]	4.2 [2.4, 5.9]	− 1.0	0.59
Sexual debut								
F	74.1 [68.7, 79.5]	65.5 [59.7, 71.2]	− 0.8	0.03	22.7 [14.8, 30.6]	17.4 [9.4, 25.4]	− 1.8	0.36
M	66.8 [56.3, 77.3]	57.6 [47, 68.2]	− 0.9	< 0.01	23.2 [19.9, 26.4]	17.6 [14.1, 21.2]	− 1.8	0.12
West Africa								
Marriage								
F	43.5 [35.5, 51.6]	35.0 [26.8, 43.1]	− 1.4	0.14	13.1 [9.0, 17.3]	9.8 [5.5, 14.0]	− 2.0	0.27
M	2.1 [1.6, 2.5]	1.8 [0.9, 2.7]	− 0.9	0.61	***	***	***	
Childbearing								
F	52.4 [45.9, 58.8]	46.9 [40.4, 53.4]	− 0.7	0.24	5.0 [3.7, 6.3]	3.9 [2.5, 5.3]	− 1.7	0.25
Sexual debut								
F	66.2 [58.1, 74.3]	57.8 [49.6, 66.0]	− 0.9	0.15	20 [16.1, 23.9]	14.6 [10.7, 18.6]	− 4.0	0.06
M	44.2 [36.9, 51.5]	29.6 [22.2, 37.0]	− 2.6	< 0.001	11.3 [8.9, 13.7]	6.2 [3.7, 8.7]	− 2.1	< 0.001
Eastern Africa								
Marriage								
F	34.9 [27.3, 42.5]	29.4 [21.3, 37.4]	− 1.1	0.33	8.4 [5.5, 11.2]	6.0 [3.1, 9.2]	− 2.2	0.27
M	5.5 [3.4, 7.5]	4.8 [2.1, 7.6]	− 0.8	0.73	***	***	***	
Childbearing								
F	48.4 [39.6, 57.2]	44.4 [35.5, 53.4]	− 0.5	0.54	3.1 [2.1, 4.1]	2.5 [1.5, 3.6]	− 1.3	0.48
Sexual debut								
F	51.8 [40.3, 63.2]	46.9 [35.5, 58.4]	− 0.6	0.57	15.0 [11.1, 18.8]	11.2 [7.2, 15.3]	− 1.9	0.19
M	48.4 [38.5, 58.3]	41.1 [31.1, 51.1]	− 1.0	< 0.001	16.7 [12.6, 20.9]	12.0 [7.7, 16.3]	− 2.2	0.12
Southern Africa								
Marriage								
F	14.6 [6.5, 22.7]	13.2 [5, 21.4]	− 0.6	0.83	1.7 [0.9, 2.6]	1.4 [0.6, 2.3]	− 1.2	0.65
M	1.3 [1.2, 1.4]	1.0 [0.7, 1.2]	− 1.8	0.01	***	***	***	
Childbearing								
F	42.5 [37.6, 47.4]	41.3 [36.1, 46.4]	− 0.1	0.76	1.5 [1.1, 1.8]	1.0 [0.5, 1.4]	− 2.8	0.08
Sexual debut								
F	47.9 [42.6, 53.2]	46.7 [40.5, 52.9]	− 0.1	0.77	7.3 [6.1, 8.4]	5.6 [4.4, 6.7]	− 1.8	0.04
M	46.7 [31.4, 62.0]	49.3 [32.3, 66.3]	0.3	< 0.01	13.3 [6.7, 19.8]	12.0 [6.0, 18.1]	− 0.6	0.80

*AARC* average annual rate of change (in percentage), and the minus sign indicates a declining trend; p-values reflect statistical significance of the absolute difference in proportion between the year 2000 and 2015; *M* male, *F* female. ***Data were not analysed due to small sample size

There are marked differences within SSA. Marriage before age 18 among girls was most common in West and Central Africa (35% and 29% respectively in 2015), and least prevalent in Southern Africa (13%). Adolescent childbearing exceeded 40% in all subregions in 2015 and was most common in Central (51%) and West Africa (47%). Sexual debut before age 18 was also more common among girls in Central and West Africa (66% and 58% respectively) than in Eastern and Southern Africa (47% in both). Among boys, marriage before age 18 occurred in less than 5% of cases in all subregions and was decreasing, while sex before age 18 was more common in Central and West Africa (58% in both) and Southern Africa (increasing to 49% in 2015).

**Fig. 2:**
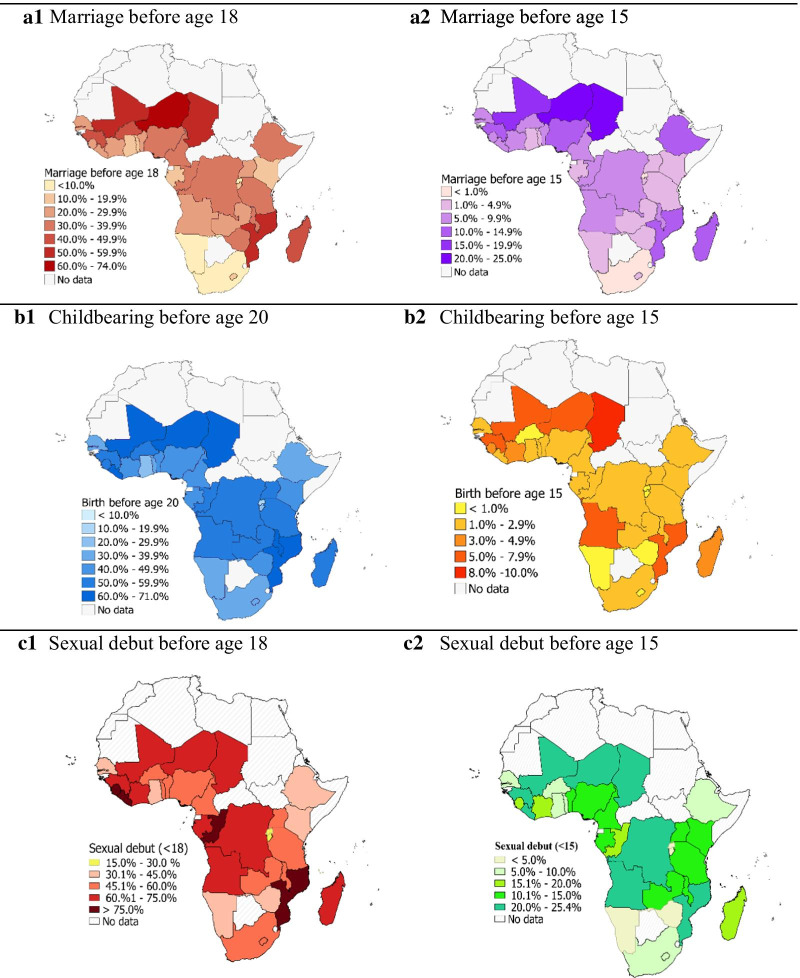
Geographical inequalities in early marriage (age 18 and 15), childbearing (age 15 and 20) and sexual debut (age 18 and 15) among adolescent girls in sub-Saharan Africa in 2015, based on national surveys conducted since 2010, sub-Saharan Africa

The subregional patterns conceal considerable variability within the subregions. Figure 2 shows the prevalence of child marriage and childbearing by country, based on the most recent national surveys. In West Africa, Mali, Niger, Burkina Faso and Guinea stand out with higher prevalence of early marriage and childbearing among adolescents, while Ghana is at the lower end in both indicators. In Central Africa, Chad and Cameroon had a more than threefold difference in both child marriage and childbearing among adolescents. In Eastern and Southern Africa, Mozambique and Zimbabwe are outliers on the higher side of both indicators, while Kenya (child marriage) and Ethiopia (childbearing) are on the lower side.

Regarding early adolescence, the prevalence of marriage, childbearing and sexual debut among girls before age 15 were estimated at 7, 3 and 12% respectively, in SSA (Table 1). For boys, only estimates for sexual debut could be made (marriage was rare), which was similar to that for girls in 2015 (12%). All indicators declined between 2000 and 2015 at an average pace of 1.6–2.3% per year, which was faster than the overall ASRH indicators.

The subregional patterns among younger adolescents are similar to the indicators that include older adolescent behaviours. In 2015, 10% and 9% of younger adolescent girls in West and Central Africa, were married before age 15, compared to 6.0% and 1.4% in Eastern and Southern Africa, respectively. Nearly 4% of girls in Central Africa had their first birth before age 15 in 2015, compared to the 2.5% in Eastern Africa and 1% in Southern Africa. All four regions showed declines during 2000–2015, most prominently for sexual debut before age 15 (both girls and boys) and marriage (girls). The average annual rate of decline was greater in the West and Central Africa than in the other subregions.

### Urban–rural inequalities

**Fig. 3:**
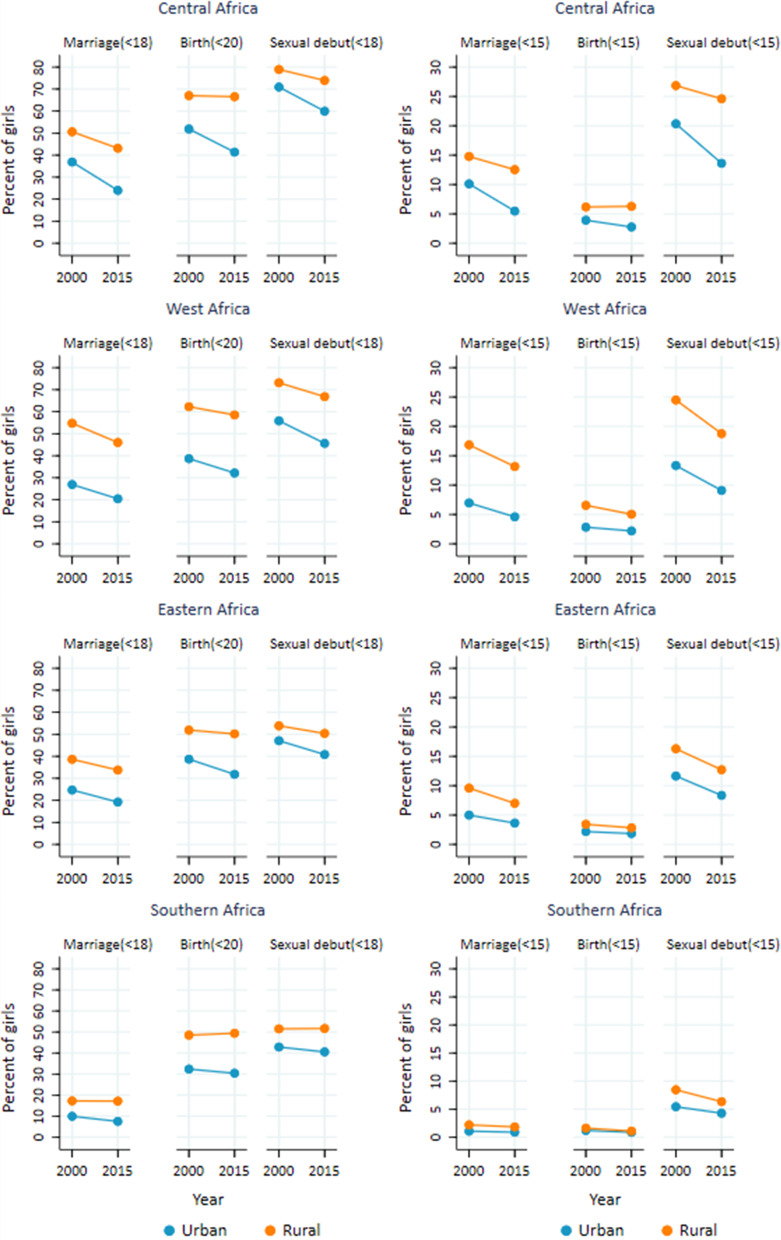
Trends in child marriage, childbearing and sexual debut among adolescent girls by subregion, **by urban–rural residence**, based on national surveys in 37 countries conducted during 1990–2018, sub-Saharan Africa

The prevalence of marriage before age 18 was almost twice as high for rural than urban girls (35% and 18% respectively). Childbearing before age 20 was also much more common (56% and 34%, respectively), as was sexual debut before age 18 (61% and 47% respectively). (Figs. 1b, 3 and Table 2). Although the rates declined during 2000–2015, the absolute urban–rural differences as well as rural to urban ratios remained either stagnant or increased overtime. The average annual reductions in marriage and births were higher for urban than rural adolescent girls.

**Table 2:** Marriage, births and sexual debut among adolescent girls between 2000 and 2015 by urban–rural residence, education and household wealth tertile, based on national surveys 1990–2018 from 37 countries, sub-Saharan Africa (in parentheses 95% confidence intervals)

	Before age 18 (20 for childbearing)				Before age 15			
	2000	2015	AARC	p	2000	2015	AARC	p
Marriage								
Overall	34.8 [25.5, 44.2]	28.0 [18.6, 37.4]	− 1.4	0.32	9.4 [5.9, 12.9]	6.7 [3.1, 10.2]	− 2.3	0.29
Rural	41.2 [28.7, 53.8]	35.0 [22.5, 47.6]	− 1.1	0.50	11.5 [6.7, 16.3]	8.7 [3.8, 13.5]	− 1.9	0.43
Urban	24.9 [17.5, 32.3]	18.1 [10.6, 25.5]	− 2.1	0.20	5.9 [3.5, 8.2]	3.8 [1.4, 6.2]	− 2.9	0.24
None or primary	43.6 [34.8, 52.4]	43.4 [34.5, 52.3]	0.0	0.98	12.0 [7.9, 16.1]	10.9 [6.7, 15]	− 0.6	0.73
Secondary +	14.7 [9.1, 20.3]	15.0 [9.4, 20.7]	0.2	0.93	2.3 [0.9, 3.7]	2.5 [1.1, 4]	0.6	0.85
Poorest (33.3%)	44.8 [32.6, 57.0]	39.8 [27.6, 52.1]	− 0.8	0.59	12.7 [7.7, 17.7]	10.2 [5.2, 15.2]	− 1.5	0.49
Middle (33.3%)	37.9 [27.3, 48.6]	29.9 [19.2, 40.6]	− 1.6	0.30	10.2 [6.4, 14]	6.9 [3.1, 10.8]	− 2.6	0.23
Richest (33.3%)	24.0 [17.8, 30.1]	15.7 [9.4, 22.0]	− 2.8	0.06	5.7 [3.6, 7.7]	3.4 [1.3, 5.5]	− 3.4	0.13
Childbearing								
Overall	50.9 [47, 54.8]	46.5 [42.5, 50.5]	− 0.6	0.12	3.8 [2.5, 5.0]	2.9 [1.7, 4.2]	− 1.6	0.38
Rural	58.0 [51.4, 64.7]	56.0 [49.3, 62.7]	− 0.2	0.70	4.7 [2.8, 6.5]	3.7 [1.8, 5.6]	− 1.5	0.50
Urban	40.7 [35.4, 46]	34.1 [28.8, 39.5]	− 1.2	< 0.001	2.6 [1.8, 3.3]	2.0 [1.3, 2.8]	− 1.6	0.31
None or primary	60.8 [56.4, 65.2]	63.6 [59.1, 68.2]	0.3	0.39	5.0 [3.5, 6.4]	4.8 [3.3, 6.3]	− 0.2	0.90
Secondary +	30.2 [23.2, 37.3]	31.2 [24.0, 38.4]	0.2	< 0.01	1.0 [0.6, 1.5]	1.1 [0.6, 1.6]	0.5	0.81
Poorest (33.3%)	61.5 [56.7, 66.3]	61.9 [56.9, 66.8]	0.0	0.92	5.4 [3.5, 7.2]	4.6 [2.8, 6.5]	− 1.0	0.59
Middle (33.3%)	54.7 [50.3, 59.2]	49.9 [45.3, 54.5]	− 0.6	< 0.001	4.0 [2.6, 5.3]	3.0 [1.6, 4.4]	− 1.8	0.35
Richest (33.3%)	37.8 [33.7, 41.9]	29.4 [25.1, 33.6]	− 1.7	0.38	2.2 [1.5, 2.9]	1.5 [0.8, 2.2]	− 2.5	0.19
Sexual debut								
Overall	60.8 [51.7, 69.8]	54.3 [45.3, 63.4]	− 0.7	0.33	16.7 [11.8, 21.7]	12.3 [7.3, 17.3]	− 2.0	0.22
Rural	65.1 [54.3, 75.9]	60.6 [49.7, 71.4]	− 0.5	0.57	19.9 [13.1, 26.6]	15.4 [8.6, 22.2]	− 1.7	0.36
Urban	54.7 [45.7, 63.7]	46.5 [37.4, 55.6]	− 1.1	< 0.001	12.8 [8.6, 17]	8.9 [4.6, 13.1]	− 2.4	0.20
None or primary	68.2 [60.3, 76.2]	67.5 [59.4, 75.5]	− 0.1	0.91	21.0 [15.7, 26.3]	18.8 [13.4, 24.1]	− 0.7	0.57
Secondary +	46.1 [35.6, 56.5]	44.1 [33.5, 54.6]	− 0.3	< 0.001	7.9 [4.7, 11.1]	7.4 [4.2, 10.6]	− 0.5	0.83
Poorest (33.3%)	67.7 [58.7, 76.6]	64.9 [55.8, 73.9]	− 0.3	0.68	21.4 [15, 27.8]	17.5 [11, 23.9]	− 1.4	0.40
Middle (33.3%)	63.3 [53.4, 73.3]	57.3 [47.2, 67.3]	− 0.7	< 0.01	17.6 [12.4, 22.8]	12.9 [7.7, 18.1]	− 2.1	0.21
Richest (33.3%)	51.8 [43.0, 60.5]	42.4 [33.6, 51.2]	− 1.3	0.37	11.8 [8.1, 15.6]	7.5 [3.7, 11.3]	− 3.1	0.11

*AARC* average annual rate of change (in percentage), and the minus sign indicates a declining trend; p-values reflect statistical significance of the absolute difference in proportion between the year 2000 and 2015. Secondary + refers to completed education level reported as secondary or higher

The subregional patterns for urban–rural residence were similar to the differences observed in SSA as a whole with rural adolescent girls at higher risk for all three events. In all four subregions and for all three indicators of adolescents, the urban AARC was greater than the rural AARC. Similarly, the absolute urban–rural difference observed in year 2000 and 2015 between urban girls and rural girls has increased for all indicators in all subregions, with the exception of child marriage in West Africa (Additional file 1: Appendix Tables S3–6). Southern Africa recorded the slowest declines for the three indicators.

In early adolescence (before age 15), 15% of rural girls and 9% of urban girls in SSA had initiated sex in 2015 (Table 2). Rural girls were more than twice as likely to be married before age 15 than urban girls (9% and 4% respectively), and almost twice as likely to give birth (4% and 2% respectively). Marriage, sexual debut and, to a lesser extent childbearing declined, reducing at an average rate of at least 1.5% per year in both rural and urban girls. Declines were observed in all four subregions in both urban and rural girls, but the relatively faster pace of decline in urban girls in all three indicators was observed in West and Central Africa.

### Wealth-related inequalities

**Fig. 4:**
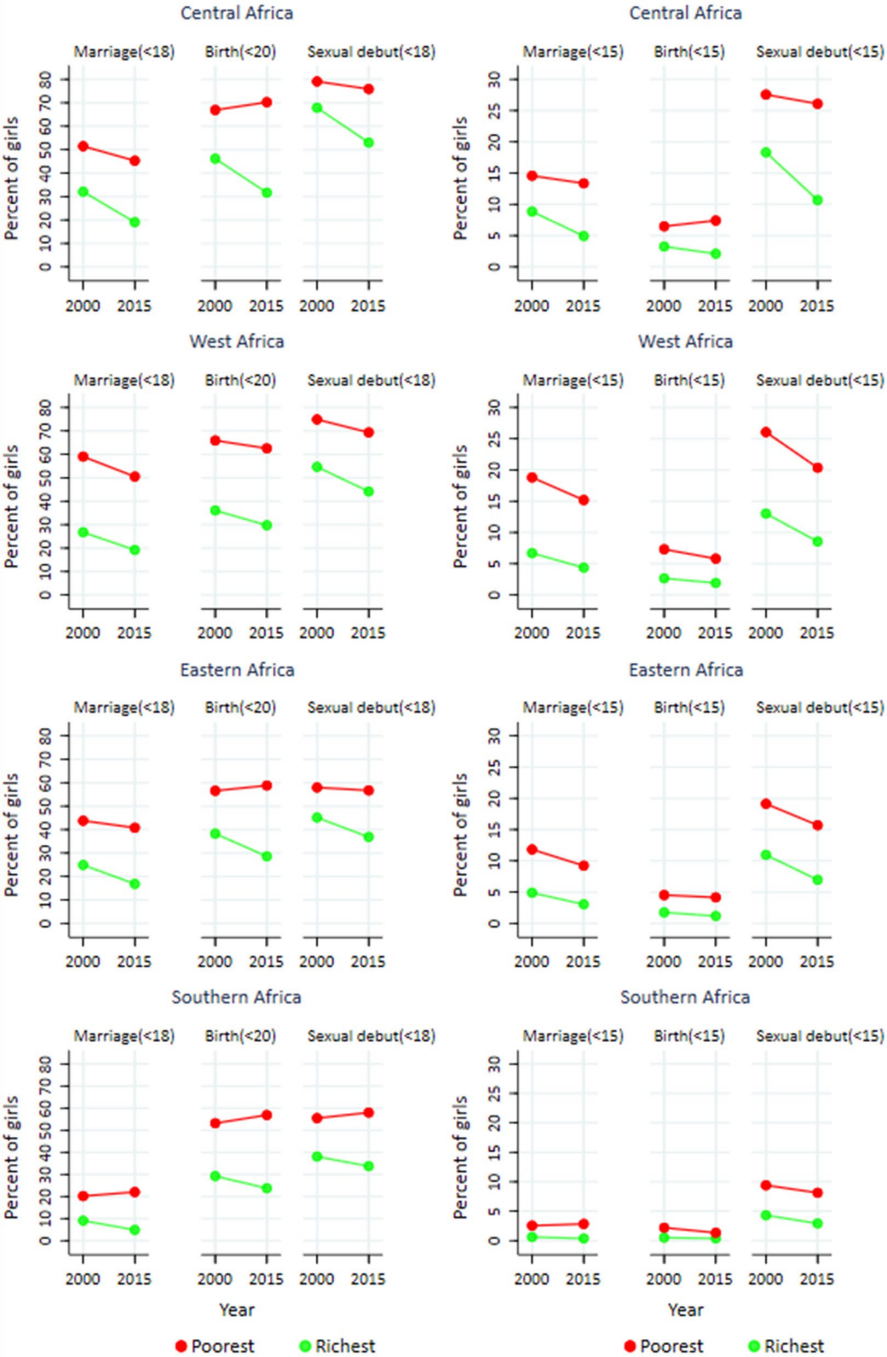
Trends in child marriage, childbearing and sexual debut among adolescent girls by subregion, **by household wealth tertile**, based on national surveys in 37 countries conducted during 1990–2018, sub-Saharan Africa

There were marked differences in ASRH indicators between the richest and poorest households in SSA. In 2015, nearly 40% of girls in the poorest tertile were married before age 18, and 62% had their first birth before age 20, compared to 16% and 29%, respectively, in the richest tertile (Figs. 1d,  4 and Table 2). There was little or no change in marriage and births among adolescent girls in the poorest tertile during 2000–2015 (AARC: -0.8% for child marriage and 0.0% for childbearing). A faster decline was observed among those in the richest tertile, with child marriage reducing at 2.8% and childbearing with 1.7% annually. Therefore, relative gaps in child marriage, adolescent birth and sexual debut between the richest and poorest tertiles in SSA expanded.

Similar patterns were observed across subregions with little progress among girls in the poorest tertile. Since girls in the richest tertile made progress on all three ASRH indicators, the gap between the poorest and richest girls widened considerably in all four subregions. The increases of the inequalities were most pronounced in Central, Eastern and West Africa for childbearing and in Central and West Africa for sexual debut.

The results for younger adolescents were similar, with girls from the poorest households experiencing higher prevalence levels and slower declines than those in the richest tertile of households. For instance, in the West Africa subregion, marriage before age 15 declined from 19 to 15% among the poorest, compared to a decline from 7 to 4% among the richest, while childbearing declined from 7 to 6% among the poorest compared to a decline from 3 to 2% among the richest, in 2000 and 2015, respectively.

### Inequalities by education

**Fig. 5:**
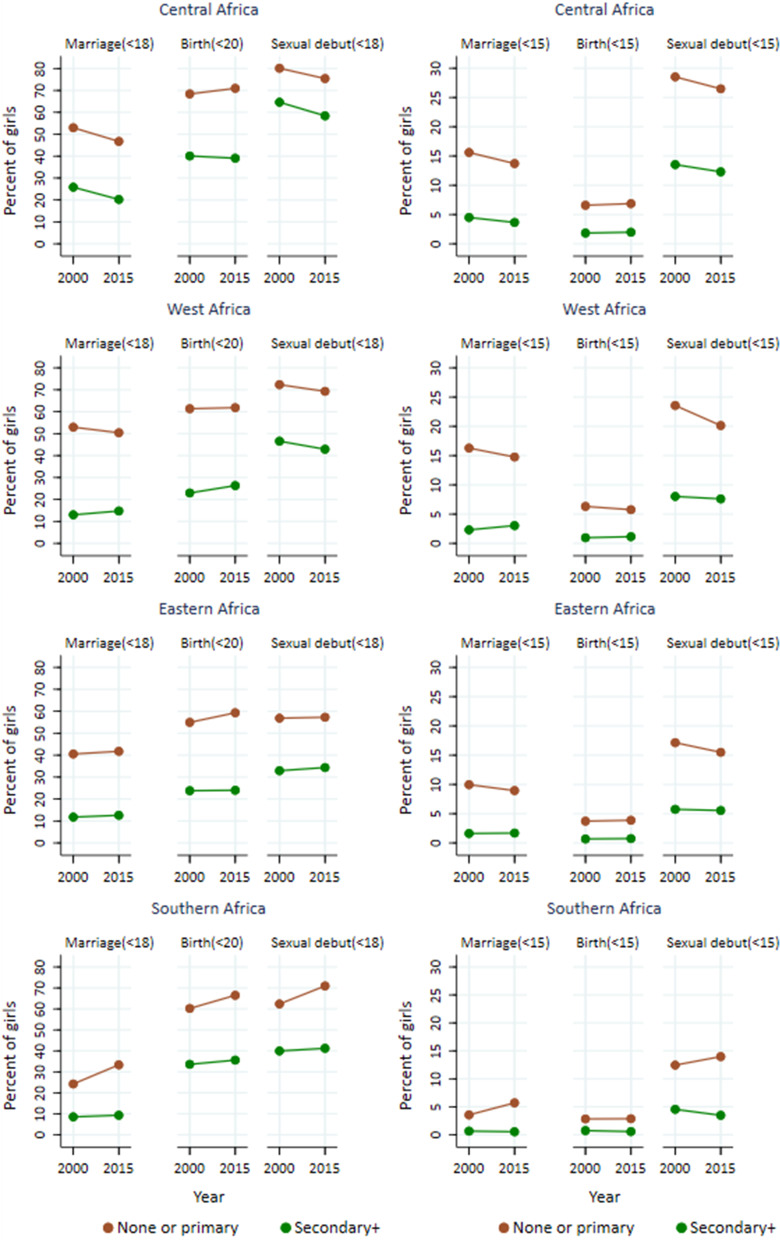
Trends in child marriage, childbearing and sexual debut among adolescent girls by subregion, **by education status**, based on national surveys in 37 countries conducted during 1990–2018, sub-Saharan Africa

The disparities in ASRH indicators for girls by education are large (Figs. 1c, 5, Table 2). In 2015, the absolute gaps between girls with primary or less education and those with secondary or higher education were 28% for child marriage, 32% for childbearing and 23% for sexual debut by age 18. Among adolescent girls with primary or less education, 43% were married by age 18 – nearly three times more than those with higher education (15%)—and 64% had their first birth by age 20, twice as high as among girls with higher education. For marriage, the gaps by education were larger than the gaps by urban—rural residence or wealth tertile, but not for the other two indicators. The trends differed from those by place of residence and wealth. There was little or no change between 2000 and 2015 within each of the two education categories in SSA. No average annual rate of change exceeded 0.3% for the three indicators.

The subregional patterns were similar to the overall pattern for SSA. The only subregion with a decline was Central Africa where the prevalence of marriage before age 18 reduced by 0.8% and 1.6% per year during 2000–2015 among those with primary or less education and secondary or more education respectively.

Girls with primary or less education are much more likely be married as well as have their first child before the age of 15 than those with secondary or higher education. In 2015, 11% of women 15–24 with primary or less education were married before age 15 and 5% of them had their first child before age 15, compared to 2.5% and 1% of women with higher education. This was consistent across all subregions, with marriage and childbearing ranging from 4% in Southern Africa to 14% in Central Africa for young women aged 15–24 with primary/no education. In general, there was no evidence of a decline in any of the three indicators of early adolescent behaviours in either education group.

## Discussion

We observed considerable progress in ASRH outcomes throughout the region of SSA, as the overall prevalence of child marriage, childbearing and sexual debut decreased during 2000–2015 for adolescent girls and boys in early and late adolescent periods. However, profound geographical disparities within and between sub-regions still persistent, and are characterized by large gaps in urban–rural differences and inequalities in socioeconomic characteristics. The increase and persistence of long-run inequalities for ASRH indicators signify the continued importance of ASRH as public health issue in the region. In particular, adolescent girls living in rural areas and in the poorest households report higher prevalence rates when compared with their more advantaged urban and richer counterparts.

Early child marriage and childbearing before age 15 have declined more markedly for younger adolescents (before age 15) than in later adolescence, which can perhaps be attributed to multi-sectoral efforts to increase girls’ access to basic education as observed over recent decades [29–32]. Our study shows, however, that in 2015 nearly 7% of girls in SSA still reported that they were married, 3% had their first birth and 12% had their first sexual intercourse before the age of 15 years. This is concerning as the risk of both maternal and infant mortality is greatest for girls aged below 15 years [9–11], and often too little resources are directed towards the younger adolescent girls [33].

Our study confirmed that the transition of ASRH is occurring across most SSA, though there are major differences between the four subregions [34–37]. In West and Central Africa marriage is more common among adolescent girls than boys and about half of the adolescent girls reported childbearing in 2015. The prevalence of child marriage in South Africa is relatively low compared to the rates reported for childbearing and sexual debut before age 18 in other subregions. Eastern Africa is often in an intermediate position. Sexual debut before age 18 followed a similar pattern for girls and boys, with the exception of boys in West Africa who had the lowest levels of the four subregions. Studies have shown that the persistence of child marriage and unplanned pregnancy in most parts of SSA are partly attributable to societal factors such as poverty, cultural norms and traditional attitudes [30, 38–40]. While many countries have made efforts to reduce child marriage, such as the adoption or reform of minimum-age-at-marriage laws and anti-child marriage campaigns [40], our results show that child marriage persists, particularly in West and Central Africa, in rural settings and among the poorest segments of the population. Analyses such as ours, that highlight where inequalities persist, may provide prevention efforts with crucial information that will allow programs to reach their intended audience.

The greater decline in child marriage compared to sexual debut before age 18 implies an increase in premarital sex: a phenomenon that has been identified and discussed in previous research for a number of sub-Saharan African countries [41]. The slower decline in childbearing compared to marriage and sexual debut among adolescent girls points to the inadequate access to modern contraceptives and other ASRH services for sexually active single adolescents as an issue in many countries with marked socioeconomic inequalities, as shown in other studies [37, 42, 43].

Early marriage and childbearing were much more common in rural than urban areas and among adolescent girls in the poorest tertile of household wealth compared to the richest tertile. These gaps persisted or even increased over time and were present in all subregions. These adverse trends highlight that the disadvantages associated with these outcomes are increasingly concentrated within already vulnerable poor and rural girls. Unequal access to education and health services are likely contributing factors [44, 45]. To make progress towards national and international goals such as the SDGs which pledge to “leaving no one behind” [46], greater attention is needed for ASRH of rural and the poorest adolescents than is currently the case.

The findings on trends by education presented a different pattern. Similar to the urban–rural and poorest-richest disparities girls with less education (primary or less) have earlier sexual debut, marriage and childbearing. Marriage and childbearing may be triggers to leaving school and also out of schoolgirls may be more likely to be married or get pregnant [8, 39, 47, 48]. The trends in the ASRH indicators during 2000–2015 however were either stable or increasing for both education categories, even though the overall trend for all girls, irrespective of educational status, declined for all three ASRH indicators. This can only be explained by a major shift in the proportion of girls from the lower to the higher education category during 2000–2015. Elsewhere, it has been shown that this change in educational attainment is indeed the main driver of changes in the age at first marriage, first sex and first birth [31, 38].

There are grave and irreversible consequences to early marriage and childbirth, affecting the social, psychological and health wellbeing of both young women and children. Child marriage is a violation of the individual’s right to make informed choices and decisions, and compromises opportunities and future prospects of the individual [49, 50]. Early initiation of sexual intercourse also elevates adolescents’ potential risk for unplanned pregnancy, abortion and STIs including HIV. Girls who become pregnant or marry early are more likely to drop out of school and this in turn limits their future opportunities.

The concerning increase in inequality for early marriage and childbearing among adolescents highlights the need for a multisectoral approach to improving ASRH. Improving all girls’ access to education and implementing gender-sensitive policies and programs that enable girls to remain in school (particularly the poorest) will foster delayed and chosen marriage and childbearing, and is critical to subsequently end early marriage and births in the region. Keeping girls in school not only reduce their risk from HIV and other STIs and unintended pregnancies [30, 51], it also contributes to their social, psychological and health wellbeing and brings opportunities that could enhance their families’ socioeconomic status in the future [30, 49, 50, 52, 53]. As far as current evidence is concerned, it is important to recognize that policies aiming to end child marriage alone could positively impact over one-third of the adolescent girls in most countries in SSA [49].

## Limitations

Our study has several limitations. We used unweighted country data for the regional and subregional regression analyses for a broad assessment of levels and trends in inequalities in SSA. Weighting by population size would likely give a somewhat different picture of the regional and subregional inequalities. We did not go into detail for specific countries, even though there are large differences between countries within SSA and even within the subregions. Country-specific analyses are needed to obtain deeper insights into inequalities in ASRH. This will also allow for analysis of within country disparities in ASRH which will be necessary for effective targeting of programs to improve ASRH.

The triple disaggregation of surveys for the analyses of ASRH indicators (by age, sex and socioeconomic characteristics), which we have presented in this paper, have received too little attention, partly due to sample size limitations. Some of the changes reached statistical significance at the 5% level but many did not due to such limitations. However, the overall picture of inequality levels and trends that emerged from our analysis was coherent and consistent for the SSA as a whole and four subregional level. This shows that it is possible to study inequalities in ASRH with DHS surveys, in spite of sample size challenges. Therefore, we recommend that future survey analyses pay greater attention to inequalities by disaggregated analyses.

Our dimensions of inequality have measurement errors. Wealth (as measured using the asset index) is recorded at time of survey, and a number of years may have elapsed between ASRH event and survey. In particular, adolescent girls who were married may have moved into a household with an economic status that differs from the household in which they grew up. For education we used the status at the time of the survey. Some respondents may still move to the higher education category by entering secondary school after the survey, but this is likely to be a small proportion at age 15 onwards.

It should also be noted that this study is limited to three ASRH indicators, each with two age cut-offs, to assess younger and older adolescent behaviours in relation to early marriage, childbearing and sexual debut. Further research is warranted to obtain greater insights into ASRH trends and inequalities, such as in age differences between spouses, sexual activity beyond first sex, contraceptive access and use, abortion, gender equity, and other critical dimensions of ASRH to inform policy and program development with effective targeting of the most vulnerable adolescents. Further studies are also required for in-depth understanding of family formation dynamics and sequencing of life events in SSA and how they vary across subregions in order to identify factors leading to disparities in geographical trends and patterns in SRH indicators.

Our analysis is based on self-reported events in the surveys, which, particularly for sexual debut, are likely to be influenced by social desirability bias [54, 55]. There is some evidence suggesting girls may deny their sexual activity, while boys may exaggerate or overstate their experiences [12]. It has also been reported that younger adolescents who have had an early first birth or marriage are more likely to overstate their age at time of survey, leading to underestimation of these events [56]. The marriage indicator also raises some methodological concerns, as in many contexts, marriage may be a process, which is difficult to date, or concepts and definitions of marriage may vary across time and populations.

## Conclusions

Early marriage and childbearing among young and older adolescent girls and boys were declining in SSA although profound subregional disparities persisted, alongside marked urban–rural and socioeconomic inequalities. Disparities between rural and urban girls, and between the poorest and richest households were increasing during 2000–2015. The widening of this gap was greater in Central and West Africa where the highest prevalence levels of child marriage and adolescent birth are found. These compound the disadvantages of already vulnerable populations.

Our study presents, to our knowledge, is one of the largest and most comprehensive assessments of inequalities in ASRH including marriage, childbearing and sexual debut among adolescents in SSA. Disaggregated data on adolescents are crucial to reach all adolescent girls and boys as part of the 2030 SDG equity agenda. The major and persistent inequalities identified in this study need to be given more attention and addressed. Our study shows that monitoring progress with national survey data is possible. Further success in the area of ASRH requires increased political will, investment, and engagement of adolescents in the process of designing and implementation of policies and programs, with much attention paid to multisectoral and life skills-oriented approaches, within the social, cultural and structural contexts.

## Supplementary Information

**Additional file 1.** Study populations and data sources, definitions, Additional Tables S1–6 and Additional Figures S1–16.

## Data Availability

All data used in the analysis for this paper are in the public domain. The Demographic and Health Surveys and AIDS Indicator Surveys can be obtained from https://www.dhsprogram.com/. Most data generated from the analysis are included in this published article and its additional files. Country specific data generated are available from the corresponding author on reasonable request.

## Abbreviations

ASRH: Adolescent sexual and reproductive health
SRH: Sexual and reproductive health
SSA: Sub-Saharan Africa
AARC: Average annual rate of change
SDGs: Sustainable development goals
HIV: Human immunodeficiency virus
AIDS: Acquired immunodeficiency syndrome
STIs: Sexually transmitted infections
DHS: Demographic and health survey
