# Supplementary material for: Inequalities in early marriage, childbearing and sexual debut among adolescents in sub-Saharan Africa
Source: Reprod Health. 2021 Jun 17;18(Suppl 1):117. doi: 10.1186/s12978-021-01125-8 (PMC8210338; doi:10.1186/s12978-021-01125-8)
Supplement: Supplementary file 1 — Additional file 1. Study populations and data sources, definitions, Additional Tables S1–6 and Additional Figures S1–16. [file 12978_2021_1125_MOESM1_ESM.pdf]

APPENDIX – supplementary materials

Study populations and data sources

Demographic and health survey data collected from adolescent and young men and women of age 15-24 were used for all analyses. These datasets are from Demographic and Health Surveys (DHS) and AIDS Indicator Surveys (AIS). A total of 129 surveys – all drawn from nationally representative household surveys – from 37 countries in sub-Saharan Africa were used for analysis. These datasets have been utilized as major source of data to produce performance and measurement indicators for low-income and middle-income countries since early 1980s. In this study, all DHS and AIS data collected between 1990-2018 were used for all analyses. For most recent survey analysis, surveys conducted between 2010-2018 were used to examine recent patterns and distributions in our variables of interest – age at first sex, marriage and birth. These datasets are publicly available and can be downloaded (https://dhsprogram.com/data/available-datasets.cfm). All datasets with subnational focus were excluded from analysis. Table S1 presents the surveys used for this study.

Table S1: Countries and surveys in sub-Saharan Africa, by region, used in the analysis

		Survey year in 5-years interval					
**Region**	**Country**	**1990-1994**	**1995-1999**	**2000--2004**	**2005-2009**	**2010-2014**	**2015-2018**
Eastern Africa	Burundi					DHS 2010	DHS 2016-17
	Comoros		DHS 1996			DHS 2012	
	Ethiopia			DHS 2000	DHS 2005	DHS 2011	DHS 2016
	Kenya		DHS 1998	DHS 2003	DHS 2008-09	DHS 2014	
	Madagascar		DHS 1997	DHS 2003-04	DHS 2008-09		
	Malawi			DHS 2000,<br>DHS 2004		DHS 2010	DHS 2015-16
	Mozambique		DHS 1997	DHS 2003	AIS 2009	DHS 2011	AIS 2015
	Rwanda	DHS 1992		DHS 2000	DHS 2005	DHS 2010	DHS 2014-15
	Tanzania	DHS 1992	DHS 1996,<br>DHS 1999	AIS 2003-04<br>DHS 2004-05	AIS 2007-08	DHS 2010,<br>AIS 2011-12	DHS 2015-16
	Uganda		DHS 1995	DHS 2000-01	DHS 2006	DHS 2011	DHS 2015-16
	Zambia			DHS 2001-02	DHS 2007	DHS 2013-14	DHS 2018
outhern Africa	Eswatini*				DHS 2006-07		
	Lesotho			DHS 2004	DHS 2009	DHS 2014	
	Namibia			DHS 2000	DHS 2006-07	DHS 2013	
	South Africa		DHS 1998				DHS 2016
	Zimbabwe		DHS 1999			DHS 2010-11	DHS 2015
Central Africa	Angola*						DHS 2015-16
	Cameroon	DHS 1991	DHS 1998	DHS 2004		DHS 2011	
	Central African Rep.*	DHS 1994-95					
	Chad		DHS 1996-97	DHS 2004		DHS 2014-15	
	Congo				DHS 2005,<br>AIS 2009	DHS 2011-12	
	DR Congo				DHS 2007	DHS 2013-14	
	Gabon			DHS 2000		DHS 2012	
	Sao Tome & Principe*				DHS 2008-09		
Western Africa	Benin		DHS 1996	DHS 2001	DHS 2006	DHS 2011-12	DHS 2017-18
	Burkina Faso	DHS 1993	DHS 1998-99	DHS 2003		DHS 2010	
	Cote d'Ivoire	DHS 1994	DHS 1998-99		AIS 2005	DHS 2011-12	
	Gambia*					DHS 2013	
	Ghana	DHS 1993	DHS 1998	DHS 2003	DHS 2008	DHS 2014	
	Guinea		DHS 1999		DHS 2005	DHS 2012	DHS 2018
	Liberia				DHS 2007	DHS 2013	
	Mali		DHS 1995-96	DHS 2001	DHS 2006	DHS 2012-13	DHS 2018
	Niger	DHS 1992	DHS 1998		DHS 2006	DHS 2012	
	Nigeria	DHS 1990		DHS 2003	DHS 2008	DHS 2013	DHS 2018
	Senegal	DHS 1992-93	DHS 1997		DHS 2005	DHS 2010-11,<br>DHS 2012-13,<br>DHS 2014	DHS 2015,<br>DHS 2016,<br>DHS 2017
	Sierra Leone				DHS 2008	DHS 2013	
	Togo		DHS 1998			DHS 2013-14	

*countries with only one Demographic and Health survey.

Data analysis

This paper is based on secondary analysis of existing data from DHS and AIS, and all analyses were done by survey for each country. The study involves mainly two components of analysis: the survival analysis done by survey for each country and linear regression analysis where data from multiple countries were pooled to describe the overall distribution of key indicators at regional and sub regional level.

Our analyses to examine inequality was informed by our conceptual framework of looking at similarities and differences by cross-cutting variables: sex of the respondent, urban vs. rural as residential area, education level and household wealth. The equity analyses involve triple disaggregation: age, sex and socioeconomic information, as well as several age-dependent indicators such as education. Education – categorized in two main groups: none or primary education as one group and secondary or higher as a second group – was based on highest level of education completed during interview[1].

Survival analysis

Survival analysis (*sts* function in the statistical package of Stata version 15) was performed to examine the distribution of early marriage, childbearing, as well as timing of sexual debut among adolescents and young men and women at time survey. The Kaplan-Meier failure function was also utilized to estimate the distribution of the cumulative probability of experiencing any of these key life events (i.e., first sex, marriage or childbirth) by each single year, particularly to estimate the distribution of early marriage, childbearing and timing of sexual debut before age 15, 18 and 20 as appropriate. For each study variable (marriage, birth and sexual debut) separately, the data were transformed into person-years in which respondents contributed the number of person-years they lived before experiencing the specific life event of interest. Reported age at first sex among those who ever had sex is set as the failure event (coded as 1) and those who never had sexual intercourse were censored (coded as 0) at their current age. The analysis replicated for the timing of first marriage and age at first childbirth.

Analysis was performed by survey for each gender (male vs. female) and country. These data were also used as underlying input data points to fit linear regression models in order to explore further the levels and trends of these key indicators at regional and sub-regional level, and assess gender gaps, urban-rural differences and inequalities in socioeconomic characteristics.

We used data from respondents 15-24 years of age, to strike the best balance between sample size and limiting the recall period for the first events of marriage, sexual debut and childbearing. With most events occurring at ages 16-18 years the longest recall period would be 6-8 years for the 24 year olds. With an average age of the sample of 15-24 year olds below 20 years, the recall period is short.

Multi-level linear regression model analysis

Multilevel mixed-effects linear regression analysis was used to fit the underlying data generated from the survival analysis in order to describe the overall distribution of key indicators at a regional and sub-regional level[2]. In order to decide the choice between fixed and random effects model, we performed Hausman test – constructed as a function of the difference between the two estimators of the model parameters to test whether the unique errors in the linear regression are correlated with time (or the independent variable or regressor) – and suggested that random-effects model fits the data better than fixed-effects model [3, 4]. For regional estimates, country-year estimates obtained from linear regression line were used to feed into the multilevel model. All analyses were performed for each sex (male vs. female), level of stratifying variable (residence: rural vs. urban, education status: primary education or less vs. secondary+, poorest vs. richest) and indicator (marriage, childbearing and sexual debut) as appropriate.

For each linear mixed model, we used maximum likelihood to fit each model, with an independent random-effects variance structure – which allows a distinct variance for each random effect within a random-effects equation and assumes that all variances are zero – implemented to specify the structure of the covariance matrix for the random effects in order to obtain best linear unbiased predictions of random effects and of conditional means (fitted values).

The multilevel data used for analysis has mainly two levels; it is characterized by a hierarchical structure, where we have 37 countries – each with multiple date points measured from 1990 to 2018 – nested within the four sub-regions (Central, Western, Southern and Eastern) and regions nested with the bigger region of sub-Saharan Africa. The subregional classifications used in the analysis are widely used by UNDP, UNICEF and other international organizations. In order to estimate the levels and trends at regional and sub regional levels, data from multiple countries in the region and each subregion were pooled to better describe the overall distribution of key indicators.

Our focus on subregions is based on the assumption of greater homogeneity (sociocultural and socioeconomic) between countries within a subregion than between subregions. In the analysis the time variable, which is the survey year in our particular case, was centered at sub-regional mean and incorporated into the random part of the model. We have assessed the fit of the models by using residual diagnostics and plots to check for heteroskedasticity, as well as looking at graphs.

Regional and country-level annual rate of changes (AARC) in percentages of key indicators were computed from established trends using linear regression models fitted to all available data from survival analysis generated using surveys between 1990-2018 (Appendix Table S1). The use of all available data and consideration to take country variations into account provides a more accurate estimate to measure the trends in marriage and childbearing among adolescents compared to using two end data points on year 2000 and 2015. The changing patterns were examined using a gender lens and relevant urban-rural and sociodemographic characteristics to shed light on the inequality of progress.

In order to assess trend levels, directions and associated p-values for urban-rural differences and inequalities by socioeconomic characteristics were obtained from time series linear regression coefficients. For each ASRH indicator and stratifying variable (identified as place of residence, education level and wealth tertile), data on differences in proportions – obtained from 37 countries of DHS data collected between 1990-2018 – used for analyses. Differences in proportions data were used as dependent variable over time (independent variable), and difference by stratifying variable were computed only between two extreme subpopulations in a stratifying variable, and that is between rural and urban for place of residence, primary or less education and secondary+ for education level, and poorest (33.3%) and richest (33.3%) populations for wealth tertile.

For each country-year estimate of prevalence of the period 2000-2015 that was used to feed into regional level estimates, as well as to compute AARC, four scenarios were considered to obtain better estimates of the period 2000-2015, and/or missing years in between:

1) if pre-2000 data were available, all data points generated from survival analysis using surveys between 1990-2000 were used as a priori information to establish trends over the period 2000-2015. Out of the 36 countries with at least one post-2000 survey, a total of 21 countries (58%) has at least one pre-2000 data point (Table S1).
2) if there was no data point available on or before 2000, the model was set to perform backward extrapolation using post-2000 surveys to obtain data point for the year 2000 and in between.
3) if there was no data point available on or after 2015, the model was set to extrapolate using all available pre-2015 data points to establish trends and obtain estimates for the period 200-2015. Out of 36 countries with at least one post-2000 survey, 16 (44%) had at least one data point available on or after 2015 (Table S1).

4) For missing data points between 2000 and 2015 (excluding the two extremes), linear regression was used to estimate the prevalence of county-year estimates for each sex, level of a stratifying variable and indicator. This method was applied to all countries with at least two surveys of which one is on or after 2009/2010.

Table S2. Early marriage, childbearing and sexual debut among adolescent girls between 2000 and 2015 by urban-rural residence, education and household wealth tertile, based on national surveys 1990-2018 from 37 countries, **sub-Saharan Africa** (in parentheses 95% confidence intervals)

		Before age 18 (20 for childbearing)					Before age 15				
		2000	2015	Diff.	AARC	P‡	2000	2015	Diff.	AAR	P‡
Marriage	Overall	34.8[25.5, 44.2]	28.0[18.6, 37.4]	-6.8	-1.4	0.32	9.4[5.9, 12.9]	6.7[3.1, 10.2]	2.7	-2.3	0.30
	(a) Rural	41.2[28.7, 53.8]	35.0[22.5, 47.6]	-6.2	-1.1	0.51	11.5[6.7, 16.3]	8.7[3.8, 13.5]	2.8	-1.9	0.43
	(b) Urban	24.9[17.5, 32.3]	18.1[10.6, 25.5]	-6.8	-2.1	0.20	5.9[3.5, 8.2]	3.8[1.4, 6.2]	2.1	-2.9	0.23
	Gap: a-b, %	16.3 (p*: 0.03)	17.0 (p*: 0.02)	0.6	0.3		5.6 (p*: 0.04)	4.8 (p*: 0.08)	-0.8	-1.0	
	Ratio: a / b, %	1.7	1.9				2.0	2.3			
	(c) None/ primary	43.6[34.8, 52.4]	43.4[34.5, 52.3]	-0.1	0.0	0.98	12.0[7.9, 16.1]	10.9[6.7, 15]	1.1	-0.6	0.73
	(d) Secondary+	14.7[9.1, 20.3]	15.0[9.4, 20.7]	0.4	0.2	0.93	2.3[0.9, 3.7]	2.5[1.1, 4]	-0.2	0.6	0.85
	Gap: c-d, (%)	28.9 (p*: <0.01)	28.4 (p*: <0.01)	-0.5	-0.1		9.7 (p*: <0.01)	8.4 (p*: <0.01)	-1.3	-1.0	
	Ratio: c/d	3.0	2.9				5.2	4.3			
	(e) Poorest (33.3%)	44.8[32.6, 57.0]	39.8[27.6, 52.1]	-4.9	-0.8	0.59	12.7[7.7, 17.7]	10.2[5.2, 15.2]	2.6	-1.5	0.49
	(f) Middle (33.3%)	37.9[27.3, 48.6]	29.9[19.2, 40.6]	-8.1	-1.6	0.30	10.2[6.4, 14]	6.9[3.1, 10.8]	3.3	-2.6	0.23
	(g) Richest (33.3%)	24.0[17.8, 30.1]	15.7[9.4, 22.0]	-8.3	-2.8	0.05	5.7[3.6, 7.7]	3.4[1.3, 5.5]	2.3	-3.4	0.13
	Gap: e-g, %	20.8 (p*: <0.01)	24.1 (p*: <0.01)	3.3	1.0		7.1 (p*: 0.01)	6.8 (p*: 0.02)	-0.3	-0.3	
	Ratio: e/g, %	1.9	2.5				2.3	3.0			
Childbearing	Overall	50.9[47, 54.8]	46.5[42.5, 50.5]	-4.4	-0.6	0.12	3.8[2.5, 5.0]	2.9[1.7, 4.2]	0.8	-1.6	0.37
	(a) Rural	58.0[51.4, 64.7]	56.0[49.3, 62.7]	-2.0	-0.2	0.70	4.7[2.8, 6.5]	3.7[1.8, 5.6]	0.9	-1.5	0.50
	(b) Urban	40.7[35.4, 46]	34.1[28.8, 39.5]	-6.6	-1.2	<0.01	2.6[1.8, 3.3]	2.0[1.3, 2.8]	0.6	-1.6	0.31
	Gap: a-b, %	17.3 (p*: <0.01)	21.9 (p*: <0.01)	4.6	1.6		2.1 (p*: 0.04)	1.7 (p*: 0.10)	-0.4	-1.3	
	Ratio: a / b, %	1.4	1.6				1.8	1.8			
	(c) None or primary	60.8[56.4, 65.2]	63.6[59.1, 68.2]	2.8	0.3	0.39	5.0[3.5, 6.4]	4.8[3.3, 6.3]	0.1	-0.2	0.90
	(d) Secondary+	30.2[23.2, 37.3]	31.2[24, 38.4]	0.9	0.2	<0.01	1.0[0.6, 1.5]	1.1[0.6, 1.6]	-0.1	0.5	0.81
	Gap: c-d, (%)	30.6 (p*: <0.01)	32.4 (p*: <0.01)	1.9	0.4		3.9 (p*: <0.01)	3.7 (p*: <0.01)	-0.2	-0.4	
	Ratio: c/d	2.0	2.0				4.8	4.3			
	(e) Poorest (33.3%)	61.5[56.7, 66.3]	61.9[56.9, 66.8]	0.4	0.0	0.92	5.4[3.5, 7.2]	4.6[2.8, 6.5]	0.7	-1.0	0.59
	(f) Middle (33.3%)	54.7[50.3, 59.2]	49.9[45.3, 54.5]	-4.8	-0.6	<0.01	4.0[2.6, 5.3]	3[1.6, 4.4]	1	-1.8	0.34
	(g) Richest (33.3%)	37.8[33.7, 41.9]	29.4[25.1, 33.6]	-8.4	-1.7	0.38	2.2[1.5, 2.9]	1.5[0.8, 2.2]	0.7	-2.5	0.19
	Gap: e-g, %	23.7 (p*: <0.01)	32.5 (p*: <0.01)	8.8	2.1		3.2 (p*: <0.01)	3.1 (p*: <0.01)	-0.1	-0.1	
	Ratio: e/g, %	1.6	2.1				2.5	3.1			
Sexual debut	Overall	60.8[51.7, 69.8]	54.3[45.3, 63.4]	-6.4	-0.7	0.33	16.7[11.8, 21.7]	12.3[7.3, 17.3]	4.4	-2.0	0.22
	(a) Rural	65.1[54.3, 75.9]	60.6[49.7, 71.4]	-4.5	-0.5	0.57	19.9[13.1, 26.6]	15.4[8.6, 22.2]	4.5	-1.7	0.36
	(b) Urban	54.7[45.7, 63.7]	46.5[37.4, 55.6]	-8.2	-1.1	<0.01	12.8[8.6, 17]	8.9[4.6, 13.1]	3.9	-2.4	0.21
	Gap: a-b, %	10.4 (p*: 0.15)	14.1 (p*: 0.04)	3.7	2.0		7.1 (p*: 0.08)	6.5 (p*: 0.11)	-0.6	-0.6	
	Ratio: a / b, %	1.2	1.3				1.6	1.7			
	(c) None or primary	68.2[60.3, 76.2]	67.5[59.4, 75.5]	-0.8	-0.1	0.91	21[15.7, 26.3]	18.8[13.4,	2.2	-0.7	0.57
	(d) Secondary+	46.1[35.6, 56.5]	44.1[33.5, 54.6]	-2.0	-0.3	<0.01	7.9[4.7, 11.1]	7.4[4.2, 10.6]	0.5	-0.5	0.83
	Gap: c-d, (%)	22.2 (p*: <0.01)	23.4 (p*: <0.01)	1.3	0.4		13.1 (p*: <0.01)	11.4 (p*:	-1.7	-0.9	
	Ratio: c/d	1.5	1.5				2.7	2.5			
	(e) Poorest (33.3%)	67.7[58.7, 76.6]	64.9[55.8, 73.9]	-2.8	-0.3	0.68	21.4[15, 27.8]	17.5[11, 23.9]	3.9	-1.4	0.40
	(f) Middle (33.3%)	63.3[53.4, 73.3]	57.3[47.2, 67.3]	-6.1	-0.7	<0.01	17.6[12.4, 22.8]	12.9[7.7, 18.1]	4.7	-2.1	0.21
	(g) Richest (33.3%)	51.8[43.0, 60.5]	42.4[33.6, 51.2]	-9.4	-1.3	0.37	11.8[8.1, 15.6]	7.5[3.7, 11.3]	4.4	-3.1	0.11
	Gap: e-g, %	15.9 (p*: 0.01)	22.5 (p*: <0.01)	6.6	2.3		9.6 (p*: 0.01)	10.0 (p*:	0.4	0.3	
	Ratio: e/g, %	1.3	1.5				1.8	2.3			

AARC = Average annual rate of change (in percentage), and the minus sign indicates a declining trend; Diff (in percentage) = Absolute difference in proportion between year 2000 and 2015. P‡ - values (row p-values) reflect statistical significance of the absolute difference in proportion between the year 2000 and 2015, while the p* - values in bracket (column p-values) reflects the statistical difference of the absolute difference between groups (urban vs. rural, education with primary or less vs. secondary+, poorest vs. richest) at year 2000 and 2015. Secondary+ refers to completed education level reported as secondary or higher.

Table S3. Patterns in urban-rural and socioeconomic characteristics in early marriage, births and sexual debut among adolescent girls between 2000 and 2015, estimates obtained from DHS surveys collected between 1990-2018 from 37 countries, **Central Africa region,** in 2000 and 2015 (in parenthesis 95% confidence intervals).

			Before age 18 (20 for childbearing)					Before age 15				
			2000	2015	Diff.	AARC	P‡	2000	2015	Diff.	AARC	P‡
Central Africa	Marriage	Overall	42.6[34.9, 50.4]	32.4[24.4, 40.4]	-10.2	-1.8	0.07	12.0[7.9, 16.2]	8.6[4.3, 12.9]	3.4	-2.2	0.27
		(a) Rural	50.6[44.2, 57.1]	43.1[36.3, 49.9]	-7.5	-1.1	0.12	14.8[10.7, 18.8]	12.5[8.2, 16.8]	2.2	-1.1	0.46
		(b) Urban	36.9[30.2, 43.5]	24.0[16.9, 31.1]	-12.9	-2.9	<0.01	10.1[6.7, 13.5]	5.5[2.0, 9.0]	4.6	-4.1	0.06
		Gap: a-b, %	13.7 (p*: <0.01)	19.1 (p*: <0.01)	5.4	2.2		4.7 (p*: 0.08)	7.0 (p*: 0.01)	2.4	2.7	
		Ratio: a / b, %	1.4	1.8				1.5	2.3			
		(c) None/primary	53.0[46.0, 60.0]	46.7[39.3, 54.2]	-6.2	-0.8	0.23	15.6[11, 20.2]	13.7[8.9, 18.5]	1.9	-0.9	0.59
		(d) Secondary+	25.8[21.2, 30.5]	20.2[14.0, 26.5]	-5.6	-1.6	0.16	4.5[2.8, 6.2]	3.6[1.1, 6.2]	0.9	-1.4	0.60
		Gap: c-d, (%)	27.1 (p*: <0.01)	26.5 (p*: <0.01)	-0.6	-0.2		11.1 (p*: <0.01)	10.1 (p*: <0.01)	-1	-0.7	
		Ratio: c/d, %	2.1	2.3				3.5	3.8			
		(e) Poorest	51.5[43.9, 59.0]	45.3[37.0, 53.6]	-6.2	-0.9	0.28	14.6[9.8, 19.4]	13.4[8.2, 18.5]	1.2	-0.6	0.75
		(f) Middle	45.2[36.6, 53.8]	34.5[25.3, 43.7]	-10.7	-1.8	0.10	12.7[7.9, 17.5]	8.2[3, 13.4]	4.5	-2.9	0.21
		(g) Richest	32.1[22.8, 41.3]	19.1[9.7, 28.4]	-13.0	-3.5	0.05	8.9[4.7, 13.0]	4.9[0.6, 9.3]	3.9	-3.9	0.20
		Gap: e-g, %	19.4 (p*: <0.01)	26.2 (p*: <0.01)	6.8	2.0		5.7 (p*: 0.08)	8.4 (p*: 0.01)	2.7	2.6	
		Ratio: e/g, %	1.6	2.4				1.6	2.7			
	Childbearing	Overall	57.8[52.9, 62.7]	51.4[46.1, 56.8]	-6.4	-0.8	0.08	4.8[3.3, 6.4]	4.2[2.4, 5.9]	0.7	-1.0	0.59
		(a) Rural	67.0[61.9, 72.1]	66.5[61.0, 72.1]	-0.5	0.0	0.91	6.2[4.4, 7.9]	6.3[4.3, 8.2]	-0.1	0.1	0.94
		(b) Urban	51.9[45.9, 57.8]	41.4[34.7, 48.1]	-10.5	-1.5	<0.01	3.9[2.7, 5.2]	2.8[1.4, 4.2]	1.1	-2.3	0.23
		Gap: a-b, %	15.1 (p*: <0.01)	25.2 (p*: <0.01)	10.0	3.4		2.3 (p*: 0.04)	3.57 (p*: <0.01)	1.3	2.9	
		Ratio: a / b, %	1.3	1.6				1.6	2.3			
		(c) None/primary	68.4[64.9, 71.8]	71.0[66.5, 75.4]	2.6	0.2	0.38	6.6[4.7, 8.4]	6.9[4.7, 9]	-0.3	0.3	0.86
		(d) Secondary+	40.1[33.8, 46.4]	39.1[31.2, 46.9]	-1.0	-0.2	<0.01	1.8[1, 2.7]	2[1, 3]	-0.1	0.5	0.85
		Gap: c-d, (%)	28.3 (p*: <0.01)	31.9 (p*: <0.01)	3.6	0.8		4.7 (p*:<0.001)	4.9 (p*: <0.01)	0.2	0.2	
		Ratio: c/d	1.7	1.8				3.6	3.5			
		(e) Poorest	66.9[62.6, 71.3]	70.2[64.8, 75.7]	3.3	0.3	0.36	6.5[4.6, 8.3]	7.4[5.2, 9.6]	-0.9	0.9	0.54
		(f) Middle	60.7[54.8, 66.6]	54.3[47.5, 61.0]	-6.4	-0.7	0.16	5.4[3.3, 7.4]	3.3[0.9, 5.8]	2	-3.2	0.21
		(g) Richest	46.2[38.7, 53.6]	31.6[23.7, 39.5]	-14.5	-2.5	<0.01	3.3[2.0, 4.5]	2.1[0.8, 3.4]	1.1	-2.9	0.21
		Gap: e-g, %	20.8 (p*: <0.01)	38.6 (p*: <0.01)	17.9	4.1		3.2 (p*: 0.004)	5.3 (p*: <0.01)	2.1	3.3	
		Ratio: e/g, %	1.4	2.2				2.0	3.5			
	Sexual debut	Overall	74.2[68.8, 79.6]	65.5[59.8, 71.3]	-8.7	-0.8	0.03	22.7[14.8, 30.6]	17.4[9.4, 25.4]	5.3	-1.8	0.36
		(a) Rural	78.9[72.7, 85.1]	73.9[67.5, 80.3]	-5.0	-0.4	0.28	26.8[21.8, 31.9]	24.6[18.4, 30.8]	2.2	-0.6	0.59
		(b) Urban	70.9[64.5, 77.4]	60.0[53.2, 66.7]	-11.0	-1.1	<0.01	20.3[17.4, 23.3]	13.6[10.5, 16.7]	6.7	-2.7	<0.01
		Gap: a-b, %	8.0 (p*: 0.08)	14.0 (p*: <0.01)	6.0	3.7		6.5 (p*: 0.030)	11.0 (p*: <0.01)	4.5	3.5	
		Ratio: a / b, %	1.1	1.2				1.3	1.8			
		(c) None/primary	80.1[75.5, 84.7]	75.4[70.4, 80.4]	-4.7	-0.4	0.17	28.5[24.4, 32.7]	26.5[21.8, 31.1]	2	-0.5	0.53
		(d) Secondary+	64.6[55.8, 73.4]	58.4[49.0, 67.8]	-6.2	-0.7	<0.01	13.5[10.8, 16.3]	12.3[9.3, 15.3]	1.2	-0.6	0.56
		Gap: c-d, (%)	15.5 (p*: <0.01)	17.0 (p*: <0.01)	1.5	0.6		15.0 (p*: <0.01)	14.2 (p*: <0.01)	-0.8	-0.4	
		Ratio: c/d	1.2	1.3				2.1	2.2			
		(e) Poorest	79.1[73.2, 85.0]	75.9[69.4, 82.3]	-3.2	-0.3	0.48	27.6[23.4, 31.8]	26.1[20.8, 31.4]	1.5	-0.4	0.68
		(f) Middle	76.6[71.6, 81.6]	69.2[63.5, 74.8]	-7.4	-0.7	0.05	23.2[18.8, 27.5]	17.4[12.2, 22.5]	5.8	-1.9	0.09
		(g) Richest	67.9[62.2, 73.5]	53.0[47.2, 58.9]	-14.9	-1.6	<0.01	18.3[15.3, 21.3]	10.7[7.5, 13.9]	7.6	-3.6	<0.01
		Gap: e-g, %	11.2 (p*: 0.01)	22.8 (p*: <0.01)	11.6	4.7		9.2 (p*: <0.01)	15.4 (p*: <0.01)	6.2	3.4	
		Ratio: e/g, %	1.2	1.4				1.5	2.4			

AARC = Average annual rate of change (in percentage), and the minus sign indicates a declining trend; Diff (in percentage) = Absolute difference in proportion between year 2000 and 2015. P‡ - values (row p-values) reflect statistical significance of the absolute difference in proportion between the year 2000 and 2015, while the p* - values in bracket (column p-values) reflects the statistical difference of the absolute difference between groups (urban vs. rural, education with primary or less vs. secondary+, poorest vs. richest) at year 2000 and 2015. Secondary+ refers to completed education level reported as secondary or higher.

Table S4. Patterns in urban-rural and socioeconomic characteristics in early marriage, childbearing and sexual debut among adolescent girls between 2000 and 2015, estimates obtained from DHS surveys collected between 1990-2018 from 37 countries, **West Africa region,** in 2000 and 2015 (in parenthesis 95% confidence intervals).

			Before age 18 (20 for childbearing)					Before age 15				
			2000	2015	Diff.	AARC	P‡	2000	2015	Diff	AARC	P‡
West Africa	Marriage	Overall	43.6[35.5, 51.6]	35.0[26.9, 43.2]	-8.6	-1.5	0.14	13.1[9.0, 17.3]	9.8[5.5, 14.0]	3.4	-2.0	0.27
		(a) Rural	54.8[46.7, 62.9]	46.0[37.8, 54.3]	-8.8	-1.2	0.14	16.9[12.1, 21.6]	13.2[8.4, 18.0]	3.7	-1.6	0.29
		(b) Urban	26.9[21.9, 31.9]	20.5[15.3, 25.6]	-6.5	-1.8	0.07	7.0[4.9, 9.0]	4.6[2.4, 6.8]	2.4	-2.8	0.12
		Gap: a-b, %	27.8 (p*: <0.01)	25.6 (p*: <0.01)	-2.3	-0.6		9.9 (p*: <0.01)	8.6 (p*: <0.01)	-1.3	-1.0	
		Ratio: a / b, %	2.0	2.3				2.4	2.9			
		(c) None/primary	52.9[44.9, 61]	50.4[42.3, 58.6]	-2.5	-0.3	0.68	16.3[11.4, 21.2]	14.8[9.8, 19.7]	1.5	-0.7	0.68
		(d) Secondary+	13.0[9.6, 16.4]	14.8[11.2, 18.3]	1.8	0.8	0.49	2.3[1.4, 3.2]	3.0[2.0, 4.0]	-0.7	1.9	0.28
		Gap: c-d, (%)	39.9 (p*: <0.01)	35.7 (p*: <0.01)	-4.3	-0.8		14.0 (p*: <0.01)	11.7 (p*: <0.01)	-2.3	-1.2	
		Ratio: c/d	4.1	3.4				7.1	4.8			
		(e) Poorest	59.0[50.9, 67.1]	50.5[42.2, 58.8]	-8.5	-1.0	0.15	18.8[13.8, 23.8]	15.2[10.1, 20.3]	3.6	-1.4	0.32
		(f) Middle	48.4[38.8, 58.0]	38.5[28.9, 48.2]	-9.9	-1.5	0.16	14.4[9.5, 19.2]	10.9[6.0, 15.7]	3.5	-1.9	0.32
		(g) Richest	26.8[19.2, 34.3]	19.2[11.6, 26.9]	-7.5	-2.2	0.17	6.7[4.0, 9.4]	4.4[1.6, 7.2]	2.3	-2.8	0.25
		Gap: e-g, %	32.3 (p*: <0.01)	31.3 (p*: <0.01)	-1.0	-0.2		12.1 (p*: <0.01)	10.8 (p*: <0.01)	-1.3	-0.8	
		Ratio: e/g, %	2.2	2.6				2.8	3.5			
	Childbearing	Overall	52.4[46.0, 58.9]	47.0[40.5, 53.5]	-5.5	-0.7	0.24	5.0[3.7, 6.3]	3.9[2.5, 5.3]	1.1	-1.7	0.25
		(a) Rural	62.3[56.3, 68.3]	58.5[52.4, 64.7]	-3.8	-0.4	0.40	6.5[4.9, 8.2]	5.0[3.3, 6.7]	1.5	-1.7	0.21
		(b) Urban	38.7[33.1, 44.3]	32.2[26.5, 37.8]	-6.5	-1.2	<0.01	2.8[2.0, 3.6]	2.2[1.4, 3.0]	0.6	-1.7	0.28
		Gap: a-b, %	23.6 (p*: <0.01)	26.3 (p*: <0.01)	2.8	0.7		3.7 (p*: <0.01)	2.8 (p*: <0.01)	-0.9	-1.8	
		Ratio: a / b, %	1.6	1.8				2.3	2.3			
		(c) None/primary	61.4[56, 66.8]	61.8[56.4, 67.3]	0.5	0.1	0.91	6.3[4.7, 8.0]	5.8[4.0, 7.5]	0.6	-0.6	0.66
		(d) Secondary+	23[18.8, 27.2]	26.3[21.3, 31.4]	3.4	0.9	<0.01	1.0[0.5, 1.4]	1.1[0.6, 1.7]	-0.2	1.2	0.63
		Gap: c-d, (%)	38.4 (p*: <0.01)	35.5 (p*: <0.01)	-2.9	-0.5		5.4 (p*: <0.01)	4.6 (p*: <0.01)	-0.7	-1	
		Ratio: c/d	2.7	2.3				6.5	5.0			
		(e) Poorest	65.9[60.4, 71.4]	62.5[57.0, 68.1]	-3.3	-0.3	0.41	7.3[5.5, 9.1]	5.8[3.9, 7.7]	1.5	-1.5	0.27
		(f) Middle	57.8[50.3, 65.3]	52.3[44.6, 60.0]	-5.5	-0.7	0.32	5.3[3.6, 7.0]	4.5[2.7, 6.2]	0.9	-1.2	0.50
		(g) Richest	36.1[28.8, 43.3]	29.7[22.3, 37.2]	-6.3	-1.3	<0.01	2.7[1.8, 3.5]	1.9[1.0, 2.8]	0.7	-2.2	0.24
		Gap: e-g, %	29.8 (p*: <0.01)	32.8 (p*: <0.01)	3.0	0.6		4.6 (p*: <0.01)	3.9 (p*: <0.01)	-0.8	-1.2	
		Ratio: e/g, %	1.8	2.1				2.7	3.0			
	Sexual debut	Overall	66.3[58.1, 74.4]	57.8[49.7, 66.0]	-8.4	-0.9	0.15	20[16.1, 23.9]	14.6[10.7, 18.6]	5.1	-4.0	0.06
		(a) Rural	73.2[66.1, 80.2]	66.8[59.6, 74.0]	-6.3	-0.6	0.22	24.5[19.9, 29]	18.8[14.1, 23.4]	5.7	-1.8	0.08
		(b) Urban	55.9[46.6, 65.1]	45.6[36.4, 54.9]	-10.2	-1.3	<0.01	13.3[10.6, 16]	9.1[6.2, 12.0]	4.2	-2.6	0.03
		Gap: a-b, %	17.3 (p*: <0.01)	21.2 (p*: <0.01)	3.9	1.4		11.2 (p*: <0.01)	9.7 (p*: <0.01)	-1.5	-0.9	
		Ratio: a / b, %	1.3	1.5				1.8	2.1			
		(c) None/primary	72.3[66.0, 78.7]	69.3[62.8, 75.8]	-3.0	-0.3	0.52	23.6[19.2, 27.9]	20.1[15.7, 24.6]	3.4	-1.0	0.29
		(d) Secondary+	46.6[36.2, 57.0]	42.9[32.4, 53.4]	-3.7	-0.5	<0.01	8.0[6.0, 10.0]	7.6[5.5, 9.7]	0.4	-0.4	0.79
		Gap: c-d, (%)	25.8 (p*: <0.01)	26.4 (p*: <0.01)	0.6	0.2		15.5 (p*: <0.01)	12.5 (p*: <0.01)	-3	-1.4	
		Ratio: c/d	1.6	1.6				2.9	2.6			
		(e) Poorest	74.9[68.0, 81.8]	69.3[62.3, 76.3]	-5.6	-0.5	0.27	26.1[21.3, 30.8]	20.4[15.6, 25.1]	5.7	-1.6	0.10
		(f) Middle	70.0[61.0, 79.0]	63.0[53.9, 72.1]	-7.0	-0.7	0.29	21.8[16.9, 26.6]	16.4[11.5, 21.3]	5.4	-1.9	0.12
		(g) Richest	54.7[44.7, 64.6]	44.2[34.2, 54.1]	-10.5	-1.4	<0.01	13.0[10.1, 15.9]	8.6[5.6, 11.5]	4.5	-2.8	0.03
		Gap: e-g, %	20.2 (p*: <0.01)	25.2 (p*: <0.01)	4.9	1.5		13.0 (p*: <0.01)	11.8 (p*: <0.01)	-1.2	-0.7	
		Ratio: e/g, %	1.4	1.6				2	2.4			

AARC = Average annual rate of change (in percentage), and the minus sign indicates a declining trend; Diff (in percentage) = Absolute difference in proportion between year 2000 and 2015. P‡ - values (row p-values) reflect statistical significance of the absolute difference in proportion between the year 2000 and 2015, while the p* - values in bracket (column p-values) reflects the statistical difference of the absolute difference between groups (urban vs. rural, education with primary or less vs. secondary+, poorest vs. richest) at year 2000 and 2015. Secondary+ refers to completed education level reported as secondary or higher.

Table S5. Patterns in urban-rural and socioeconomic characteristics in child marriage, adolescent birth and sexual debut among adolescent girls between 2000 and 2015, estimates obtained from DHS surveys collected between 1990-2018 from 37 countries, **Eastern Africa region,** in 2000 and 2015 (in parenthesis 95% confidence intervals).

			Before age 18 (20 for childbearing)					Before age 15				
			2000	2015	Diff.	AARC	P‡	2000	2015	Diff.	AARC	P‡
Eastern Africa	Marriage	Overall	34.9[27.3, 42.6]	29.4[21.3, 37.5]	-5.5	-1.1	0.57	8.4[5.5, 11.2]	6.0[3.0, 9.0]	2.4	-2.2	0.27
		(a) Rural	38.6[29.7, 47.6]	33.8[24.5, 43.0]	-4.9	-0.9	0.71	9.6[6.2, 13.0]	7.0[3.4, 10.6]	2.6	-2.1	0.31
		(b) Urban	24.7[19.8, 29.6]	19.2[13.7, 24.8]	-5.5	-1.7	<0.01	5.0[3.6, 6.4]	3.6[2.1, 5.2]	1.4	-2.1	0.21
		Gap: a-b, %	13.9 (p*: 0.01)	14.5 (p*: <0.01)	0.6	0.3		4.6 (p*: 0.02)	3.4 (p*: 0.09)	-1.2	-2.1	
		Ratio: a / b, %	1.6	1.8				1.9	1.9			
		(c) None/primary	40.5[31.5, 49.6]	41.8[32.1, 51.4]	1.2	0.2	0.96	10[6.6, 13.4]	9.0[5.3, 12.6]	1.0	-0.7	0.70
		(d) Secondary+	11.8[8.0, 15.6]	12.6[8.1, 17.2]	0.8	0.5	<0.01	1.6[0.9, 2.4]	1.7[0.8, 2.6]	-0.1	0.3	0.91
		Gap: c-d, (%)	28.7 (p*: <0.01)	29.2 (p*: <0.01)	0.4	0.1		8.3 (p*: <0.01)	7.3 (p*: <0.01)	-1.1	-0.9	
		Ratio: c/d	3.4	3.3				6.1	5.3			
		(e) Poorest	43.8[34.1, 53.5]	40.8[30.9, 50.7]	-3.0	-0.5	0.90	11.8[7.7, 15.9]	9.2[5, 13.4]	2.6	-1.7	0.39
		(f) Middle	38.1[29.1, 47.0]	30.9[21.5, 40.3]	-7.1	-1.4	0.54	9.1[5.7, 12.5]	5.8[2.3, 9.4]	3.3	-3.0	0.19
		(g) Richest	24.9[19.7, 30.1]	16.8[10.6, 23.1]	-8.1	-2.6	<0.01	4.9[3.2, 6.6]	3.1[1.1, 5.0]	1.8	-3.2	0.16
		Gap: e-g, %	18.9 (p*: <0.01)	24.0 (p*: <0.01)	5.1	1.6		6.9 (p*: <0.01)	6.2 (p*: <0.01)	-0.7	-0.8	
		Ratio: e/g, %	1.8	2.4				2.4	3.0			
	Childbearing	Overall	48.4[39.6, 57.3]	44.5[35.5, 53.4]	-4.0	-0.6	0.55	3.1[2.1, 4.1]	2.5[1.5, 3.6]	0.5	-1.3	0.48
		(a) Rural	51.9[42.0, 61.9]	50.2[40.1, 60.2]	-1.7	-0.2	0.82	3.4[2.2, 4.7]	2.8[1.6, 4.1]	0.6	-1.3	0.52
		(b) Urban	38.7[31.2, 46.2]	31.8[24.2, 39.4]	-6.9	-1.3	<0.01	2.2[1.6, 2.8]	1.8[1.2, 2.5]	0.3	-1.1	0.47
		Gap: a-b, %	13.2 (p*: 0.04)	18.4 (p*: <0.01)	5.2	2.2		1.2 (p*: 0.08)	1.0 (p*: 0.18)	-0.3	-1.5	
		Ratio: a / b, %	1.3	1.6				1.6	1.5			
		(c) None/primary	54.9[45.2, 64.7]	59.3[49.3, 69.3]	4.4	0.5	0.55	3.7[2.4, 5.1]	3.9[2.5, 5.2]	-0.1	0.3	0.89
		(d) Secondary+	23.8[17.1, 30.5]	24.0[17.1, 30.9]	0.2	0.1	<0.01	0.7[0.4, 1.0]	0.7[0.3, 1.2]	-0.1	0.1	0.82
		Gap: c-d, (%)	31.1 (p*: <0.01)	35.3 (p*: <0.01)	4.2	0.8		3.0 (p*: <0.01)	3.1 (p*: <0.01)	0.1	0.2	
		Ratio: c/d	2.3	2.5				5.5	5.2			
		(e) Poorest	56.6[46.3, 66.8]	58.8[48.5, 69.2]	2.2	0.3	0.78	4.5[2.7, 6.4]	4.2[2.3, 6.0]	0.4	-0.6	0.79
		(f) Middle	51.8[42.1, 61.5]	47.1[37.3, 56.9]	-4.7	-0.6	0.51	3.2[2.0, 4.3]	2.4[1.2, 3.6]	0.8	-1.9	0.36
		(g) Richest	38.3[30.8, 45.8]	28.6[20.8, 36.4]	-9.7	-1.9	<0.01	1.8[1.3, 2.2]	1.2[0.7, 1.7]	0.6	-2.7	0.10
		Gap: e-g, %	18.3 (p*: <0.01)	30.2 (p*: <0.01)	11.9	3.3		2.8 (p*: <0.01)	3.0 (p*: <0.01)	0.2	0.5	
		Ratio: e/g, %	1.5	2.1				2.6	3.5			
	Sexual debut	Overall	51.8[40.4, 63.2]	47.0[35.5, 58.5]	-4.8	-0.6	0.33	15.0[11.1, 18.8]	11.2[7.2, 15.3]	3.7	-1.9	0.19
		(a) Rural	53.9[41.6, 66.1]	50.4[38.1, 62.7]	-3.5	-0.4	0.47	16.3[11.7, 20.8]	12.7[8.0, 17.4]	3.6	-1.7	0.28
		(b) Urban	47.1[37.0, 57.2]	40.8[30.5, 51.2]	-6.3	-1.0	0.15	11.6[9.0, 14.3]	8.4[5.5, 11.2]	3.3	-2.2	0.10
		Gap: a-b, %	6.8 (p*: 0.67)	9.6 (p*: 0.25)	2.8	2.3		4.6 (p*: 0.09)	4.4 (p*: 0.02)	-0.3	-0.4	
		Ratio: a / b, %	1.1	1.2				1.4	1.5			
		(c) None/primary	56.9[44.7, 69.0]	57.3[45.0, 69.5]	0.4	0.0	0.86	17.1[12.6, 21.7]	15.5[10.8, 20.2]	1.7	-0.7	0.63
		(d) Secondary+	32.9[22.5, 43.3]	34.4[23.9, 44.8]	1.4	0.3	0.80	5.7[4.1, 7.4]	5.5[3.8, 7.3]	0.2	-0.2	0.87
		Gap: c-d, (%)	23.9 (p*: <0.01)	22.9 (p*: <0.01)	-1.0	-0.3		11.4 (p*: <0.01)	9.9 (p*: <0.01)	-1.5	-0.9	
		Ratio: c/d	1.7	1.7				3.0	2.8			
		(e) Poorest	58.0[45.2, 70.8]	56.7[43.8, 69.6]	-1.2	-0.1	0.69	19.1[13.7, 24.6]	15.7[10.1, 21.3]	3.4	-1.3	0.40
		(f) Middle	53.6[41.5, 65.8]	48.1[35.9, 60.3]	-5.6	-0.7	0.28	15.5[11.1, 19.9]	11.3[6.8, 15.8]	4.2	-2.1	0.19
		(g) Richest	45.1[34.9, 55.4]	36.9[26.4, 47.4]	-8.3	-1.4	0.05	11[8.3, 13.6]	7.0[4.0, 9.9]	4	-3.0	0.05
		Gap: e-g, %	12.8 (p*: 0.13)	19.9 (p*: 0.02)	7.1	2.9		8.2 (p*:<0.01)	8.7 (p*: <0.01)	0.6	0.5	
		Ratio: e/g, %	1.3	1.5				1.7	2.3			

AARC = Average annual rate of change (in percentage), and the minus sign indicates a declining trend; Diff (in percentage) = Absolute difference in proportion between year 2000 and 2015. P‡ - values (row p-values) reflect statistical significance of the absolute difference in proportion between the year 2000 and 2015, while the p* - values in bracket (column p-values) reflects the statistical difference of the absolute difference between groups (urban vs. rural, education with primary or less vs. secondary+, poorest vs. richest) at year 2000 and 2015. Secondary+ refers to completed education level reported as secondary or higher.

Table S6. Patterns in urban-rural and socioeconomic characteristics in child marriage, adolescent birth and sexual debut among adolescent girls between 2000 and 2015, estimates obtained from DHS surveys collected between 1990-2018 from 37 countries, **Southern Africa region,** in 2000 and 2015 (in parenthesis 95% confidence intervals).

			Before age 18 (20 for childbearing)					Before age 15				
			2000	2015	Diff.	AARC	P‡	2000	2015	Diff.	AARC	P‡
Southern Africa	Marriage	Overall	14.7[6.6, 22.8]	13.3[5.1, 21.5]	-1.4	-0.7	0.83	1.7[0.9, 2.6]	1.4[0.6, 2.3]	0.3	-1.2	0.65
		(a) Rural	17.2[7.2, 27.3]	17.1[6.8, 27.5]	-0.1	0.0	0.99	2.2[0.9, 3.5]	1.8[0.6, 3.1]	0.4	-1.2	0.72
		(b) Urban	9.9[4.9, 14.9]	7.5[2.5, 12.6]	-2.4	-1.9	0.52	1.1[0.8, 1.4]	0.9[0.6, 1.2]	0.2	-1.3	0.44
		Gap: a-b, %	7.3 (p*: 20)	9.6 (p*: 0.10)	2.3	1.8		1.1 (p*: 0.10)	0.9 (p*: 0.16)	-0.2	-1.1	
		Ratio: a / b, %	1.7	2.3				2.0	2.1			
		(c) None/primary	24.2[12.1, 36.4]	33.3[21.2, 45.5]	9.1	2.1	0.30	3.6[1.2, 5.9]	5.7[3.3, 8.1]	-2.1	3.2	0.22
		(d) Secondary+	8.5[2.7, 14.3]	9.3[3.2, 15.3]	0.8	0.6	0.87	0.7[0.3, 1]	0.5[0.2, 0.9]	0.1	-1.2	0.64
		Gap: c-d, (%)	15.7 (p*: 0.02)	24.0 (p*: <0.01)	8.4	2.8		2.9 (p*: 0.02)	5.2 (p*: <0.01)	2.3	3.8	
		Ratio: c/d	2.8	3.6				5.4	10.5			
		(e) Poorest	20.2[8.5, 31.9]	22[9.8, 34.2]	1.8	0.6	0.84	2.6[1.0, 4.1]	2.8[1.3, 4.4]	-0.3	0.7	0.81
		(f) Middle	14.8[5.7, 23.9]	13.8[4.6, 23.0]	-1.0	-0.5	0.89	1.9[1.0, 2.9]	1.3[0.3, 2.3]	0.6	-2.6	0.38
		(g) Richest	9.1[5.2, 13.0]	4.9[1.3, 8.5]	-4.2	-4.1	0.12	0.6[0.4, 0.8]	0.4[0.1, 0.6]	0.3	-3.4	0.12
		Gap: e-g, %	11.1 (p*: 0.08)	17.1 (p*: <0.01)	6.0	2.9		1.9 (p*: 0.01)	2.5 (p*: <0.01)	0.5	1.6	
		Ratio: e/g, %	2.2	4.5				4.1	7.5			
	Childbearing	Overall	42.5[37.6, 47.4]	41.3[36.1, 46.5]	-1.2	-0.2	0.76	1.5[1.1, 1.8]	1.0[0.5, 1.4]	0.5	-2.8	0.08
		(a) Rural	48.5[43.2, 53.8]	49.4[43.3, 55.5]	0.9	0.1	0.84	1.6[1.2, 2.1]	1.1[0.5, 1.7]	0.5	-2.6	0.15
		(b) Urban	32.4[28.7, 36.0]	30.4[26.6, 34.2]	-2.0	-0.4	<0.01	1.2[0.6, 1.7]	0.9[0.3, 1.4]	0.3	-2.1	0.42
		Gap: a-b, %	16.1 (p*: <0.01)	19.0 (p*: <0.01)	2.9	1.1		0.4 (p*: 0.25)	0.2 (p*: 0.60)	-0.2	-4.3	
		Ratio: a / b, %	1.5	1.6				1.4	1.3			
		(c) None/primary	60.2[53.2, 67.3]	66.5[58.1, 74.9]	6.3	0.7	0.27	2.8[1.9, 3.8]	2.9[1.7, 4.0]	0	0.1	0.96
		(d) Secondary+	33.6[29.4, 37.8]	35.6[31.2, 40.0]	2.0	0.4	<0.01	0.8[0.4, 1.1]	0.6[0.2, 0.9]	0.2	-1.8	0.46
		Gap: c-d, (%)	26.6 (p*: <0.01)	30.9 (p*: <0.01)	4.3	1.0		2.1 (p*: <0.01)	2.3 (p*:<0.01)	0.2	0.7	
		Ratio: c/d	1.8	1.9				3.8	5.0			
		(e) Poorest	53.2[47.2, 59.1]	56.9[49.7, 64.0]	3.7	0.4	0.44	2.2[1.7, 2.7]	1.3[0.7, 2.0]	0.9	-3.3	0.05
		(f) Middle	43.5[38.4, 48.6]	43.1[37.5, 48.7]	-0.4	-0.1	0.92	1.4[1.0, 1.9]	1.0[0.5, 1.4]	0.5	-2.5	0.15
		(g) Richest	29.2[23.2, 35.3]	23.7[17.5, 30.0]	-5.5	-1.4	<0.01	0.5[0.3, 0.8]	0.4[0.0, 0.7]	0.1	-2.2	0.50
		Gap: e-g, %	23.9 (p*: <0.01)	33.1 (p*: <0.01)	9.2	2.2		1.7 (p*: <0.01)	1.0 (p*: 0.01)	-0.7	-3.7	
		Ratio: e/g, %	1.8	2.4				4.2	3.6			
	Sexual debut	Overall	48[42.7, 53.3]	46.7[40.5, 52.9]	-1.3	-0.2	0.77	7.3[6.1, 8.4]	5.6[4.4, 6.7]	1.7	-1.8	0.04
		(a) Rural	51.5[46.6, 56.3]	51.7[45.9, 57.4]	0.2	0.0	0.96	8.4[7.4, 9.5]	6.3[5.3, 7.4]	2.1	-1.9	<0.01
		(b) Urban	42.9[35, 50.8]	40.5[32.5, 48.5]	-2.3	-0.4	<0.01	5.4[3.8, 7.1]	4.3[2.6, 5.9]	1.2	-1.6	0.33
		Gap: a-b, %	8.6 (p*: 0.07)	11.1 (p*: 0.03)	2.5	1.7		3.0 (p*: 0.002)	2.1 (p*: 0.04)	-1	-2.5	
		Ratio: a / b, %	1.2	1.3				1.6	1.5			
		(c) None/primary	62.4[57.4, 67.3]	70.9[65.7, 76.2]	8.6	0.9	0.02	12.5[10.4, 14.5]	14.0[11.9, 16.1]	-1.5	0.8	0.32
		(d) Secondary+	40.0[32.4, 47.6]	41.2[32.2, 50.3]	1.2	0.2	<0.01	4.5[3.3, 5.8]	3.5[2.2, 4.8]	1.1	-1.8	0.25
		Gap: c-d, (%)	22.4 (p*: <0.01)	29.7 (p*: <0.01)	7.3	1.9		7.9 (p*: <0.01)	10.5 (p*: <0.01)	2.6	1.9	
		Ratio: c/d	1.6	1.7				2.7	4.0			
		(e) Poorest	55.5[51.3, 59.6]	58.0[53.0, 63.0]	2.5	0.3	0.46	9.4[8.3, 10.5]	8.1[7.0, 9.3]	1.3	-1	0.12
		(f) Middle	49.7[44.4, 55.0]	48.1[41.6, 54.6]	-1.6	-0.2	0.72	7.7[6.3, 9.1]	5.4[4.0, 6.8]	2.3	-2.4	0.02
		(g) Richest	38.1[30.9, 45.2]	33.7[26.3, 41.2]	-4.3	-0.8	<0.01	4.3[2.8, 5.8]	2.9[1.3, 4.6]	1.4	-2.6	0.22
		Gap: e-g, %	17.4 (p‡: <0.01)	24.2 (p*: <0.01)	6.9	2.2		5.1 (p*: <0.01)	5.2 (p*: <0.01)	0.1	0.2	
		Ratio: e/g, %	1.5	1.7				2.2	2.8			

AARC = Average annual rate of change (in percentage), and the minus sign indicates a declining trend; Diff (in percentage) = Absolute difference in proportion between year 2000 and 2015. P‡ - values (row p-values) reflect statistical significance of the absolute difference in proportion between the year 2000 and 2015, while the p* - values in bracket (column p-values) reflects the statistical difference of the absolute difference between groups (urban vs. rural, education with primary or less vs. secondary+, poorest vs. richest) at year 2000 and 2015. Secondary+ refers to completed education level reported as secondary or higher.

Figure S1. Levels and trends of child marriage (age 18), childbearing (age 20) and sexual debut (age 18) among adolescent girls and boys sub-Saharan Africa (early child marriage and birth (age 15) among early adolescent boys was found low and are not included in the figure)

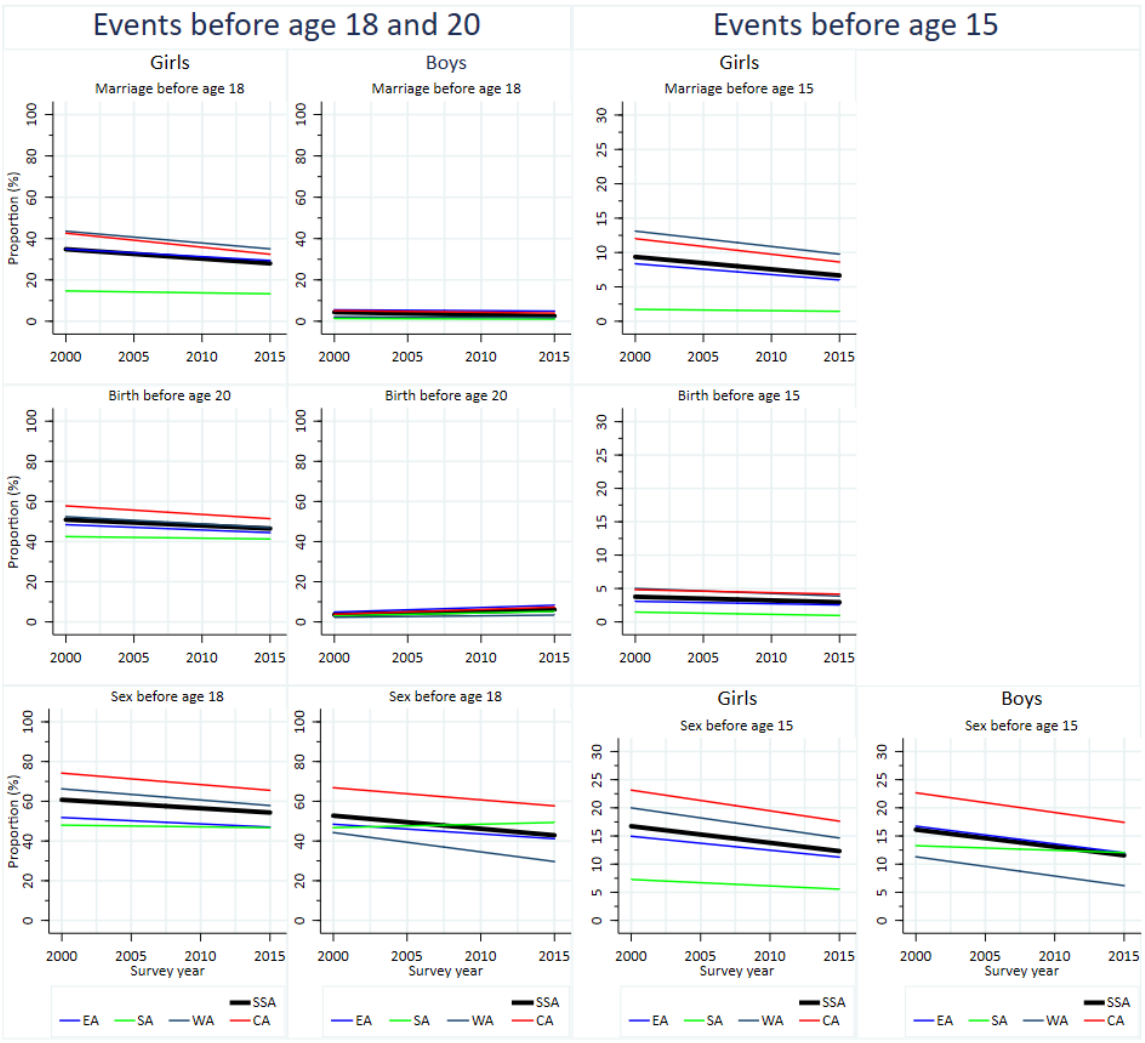

SSA=sub-Saharan Africa; EA=Eastern Africa; SA=Southern Africa; CA=Central Africa; WA=Western Africa.
Source: Demographic and Health Surveys data collected between 1990-2018.

Figure S2. Levels and trends child marriage (age 18), childbearing (age 20) and sexual debut (age 18) among adolescent girls in sub-Saharan Africa, disaggregated by place of residence, education and wealth tertile

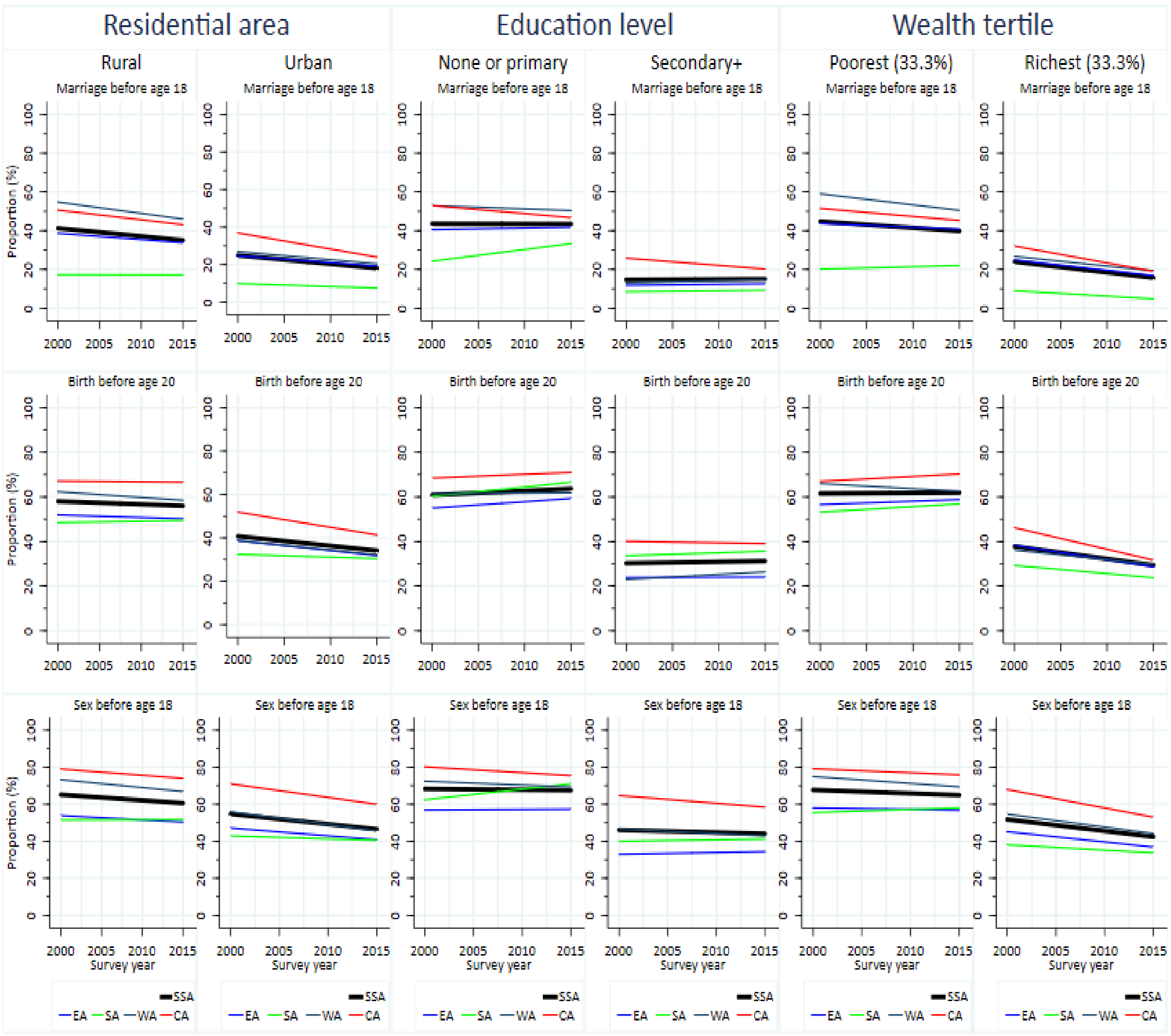

SSA=sub-Saharan Africa; EA=Eastern Africa; SA=Southern Africa; CA=Central Africa; WA=Western Africa.
Source: Demographic and Health Surveys data collected between 1990-2018.

Figure S3. Levels and trends of early marriage (age 15), childbearing (age 15) and sexual debut (age 15) among adolescent girls, in sub-Saharan Africa, disaggregated by place of residence, education and wealth tertile

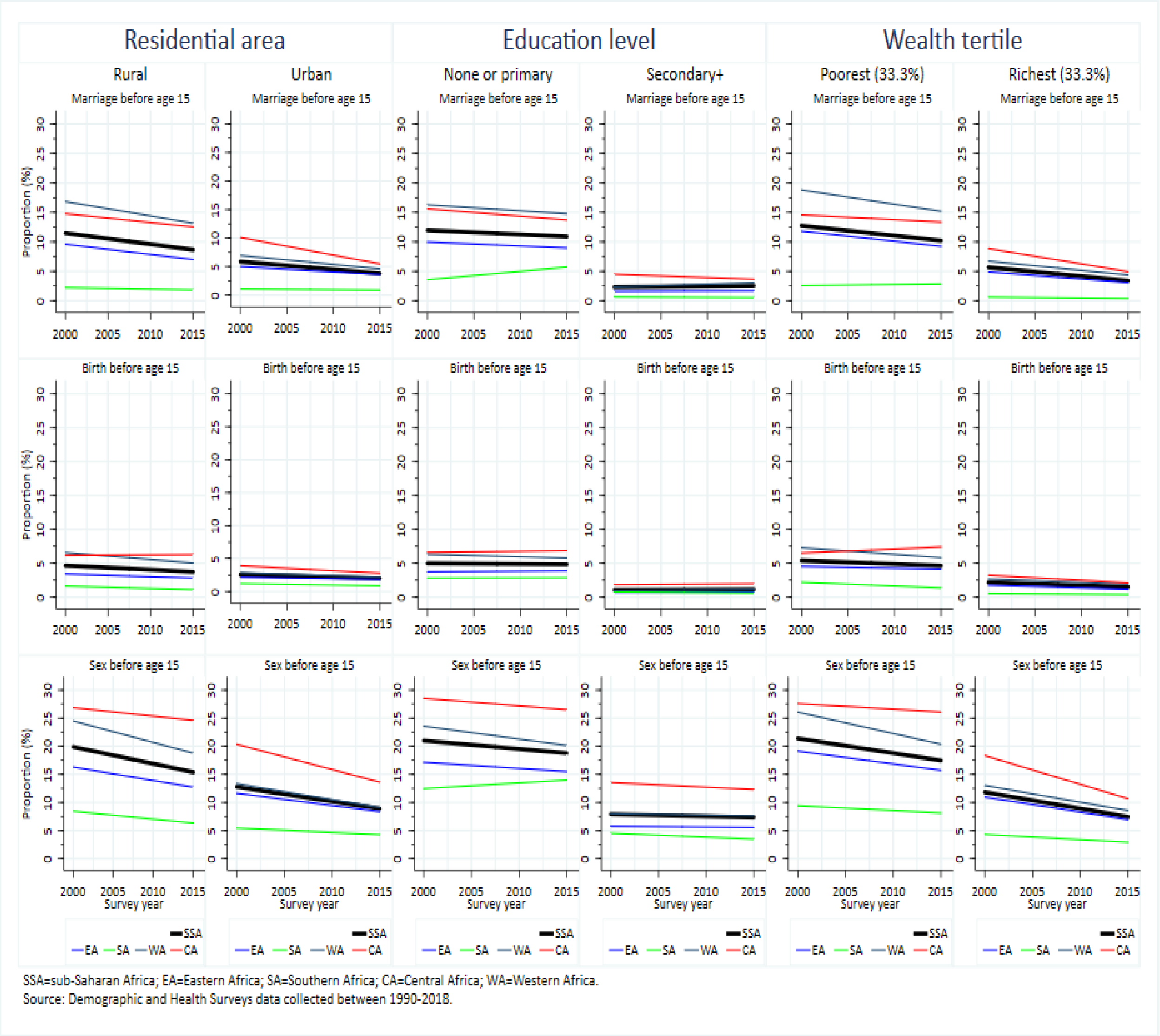

Figure S4. Percentages of countries with significant reduction overtime(p<0.05): adolescent girls child marriage, childbearing, and sexual debut, 32 countries with at least two surveys, sub-Saharan Africa.

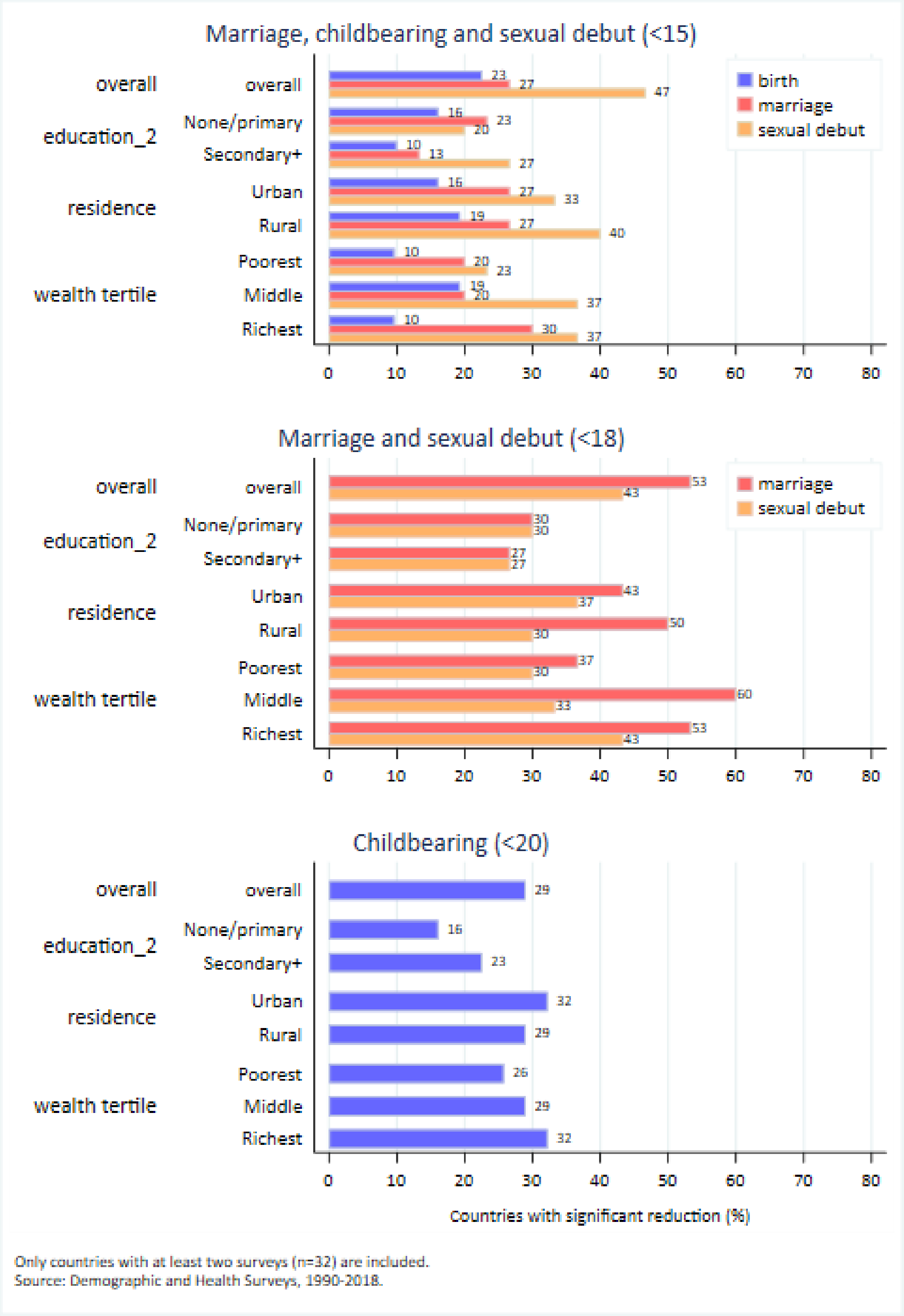

Figure S5. Overall trends in urban-rural differences and inequalities by socioeconomic characteristics in the proportions of adolescent girls' child marriage, childbearing and sexual debut, 37 countries, sub-Saharan Africa.

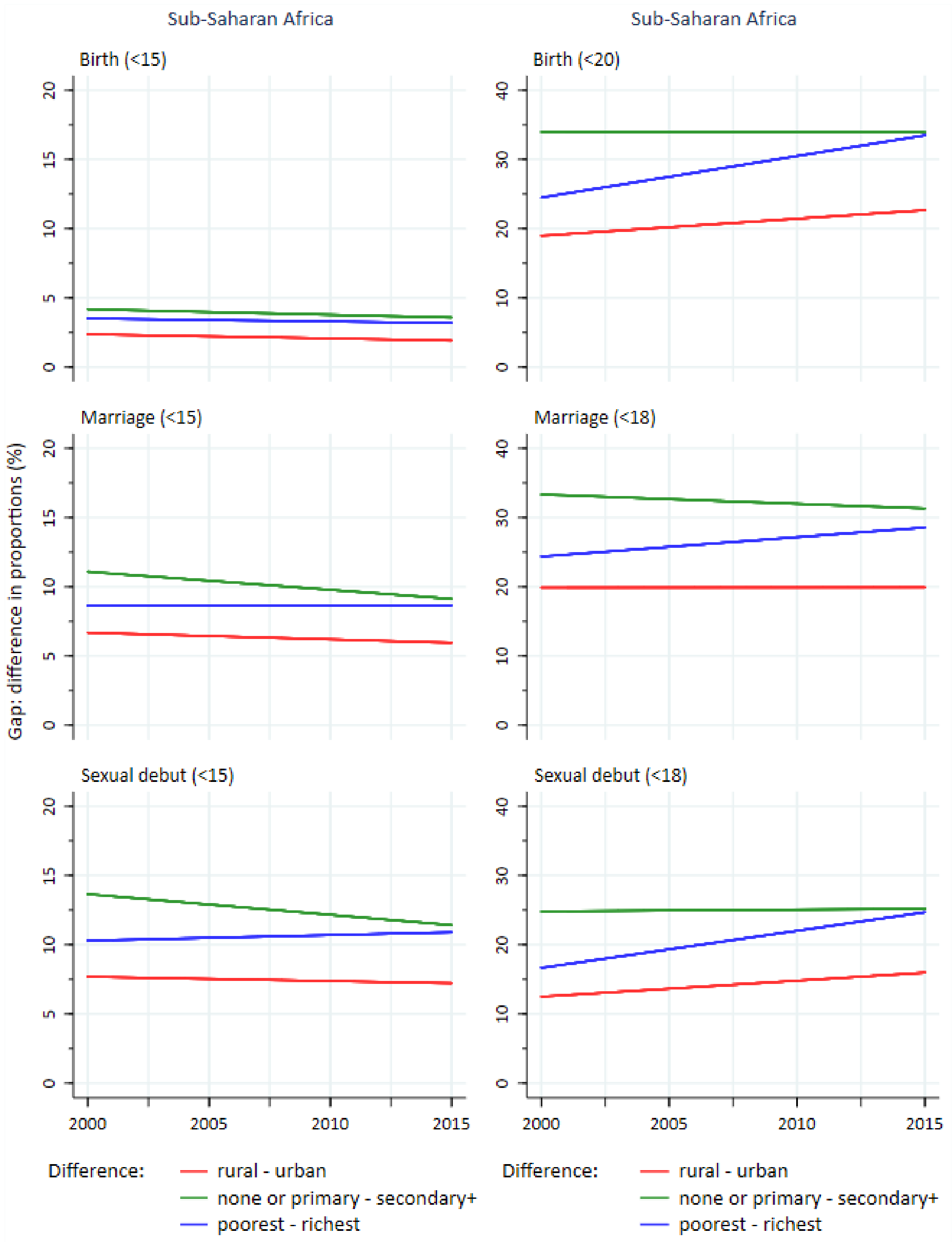

Poorest and richest classification reflects wealth tertile, each representing 33.3%
Source: Demographic and Health Surveys, 1990-2018

Figure S6. Trends in urban-rural differences and gaps in socioeconomic characteristics in the proportions of adolescent girls' child marriage (age 18), childbearing (age 20) and sexual debut (age 18), 37 countries, sub-Saharan Africa.

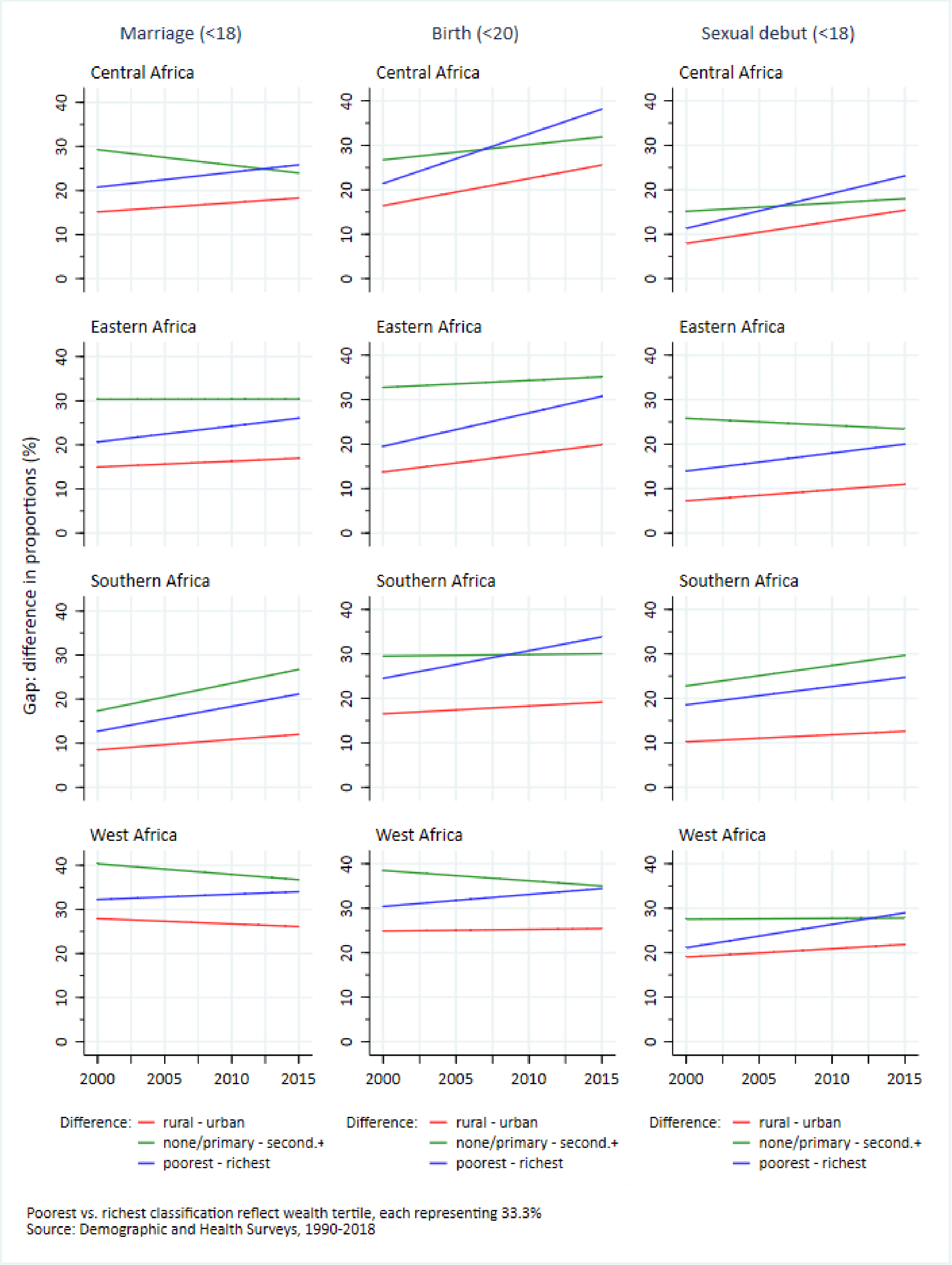

Figure S7. Levels and trends in urban-rural differences and gaps in socioeconomic characteristics in the proportion of adolescent girls' child marriage, childbearing and sexual debut before age 15, 37 countries, sub-Saharan Africa.

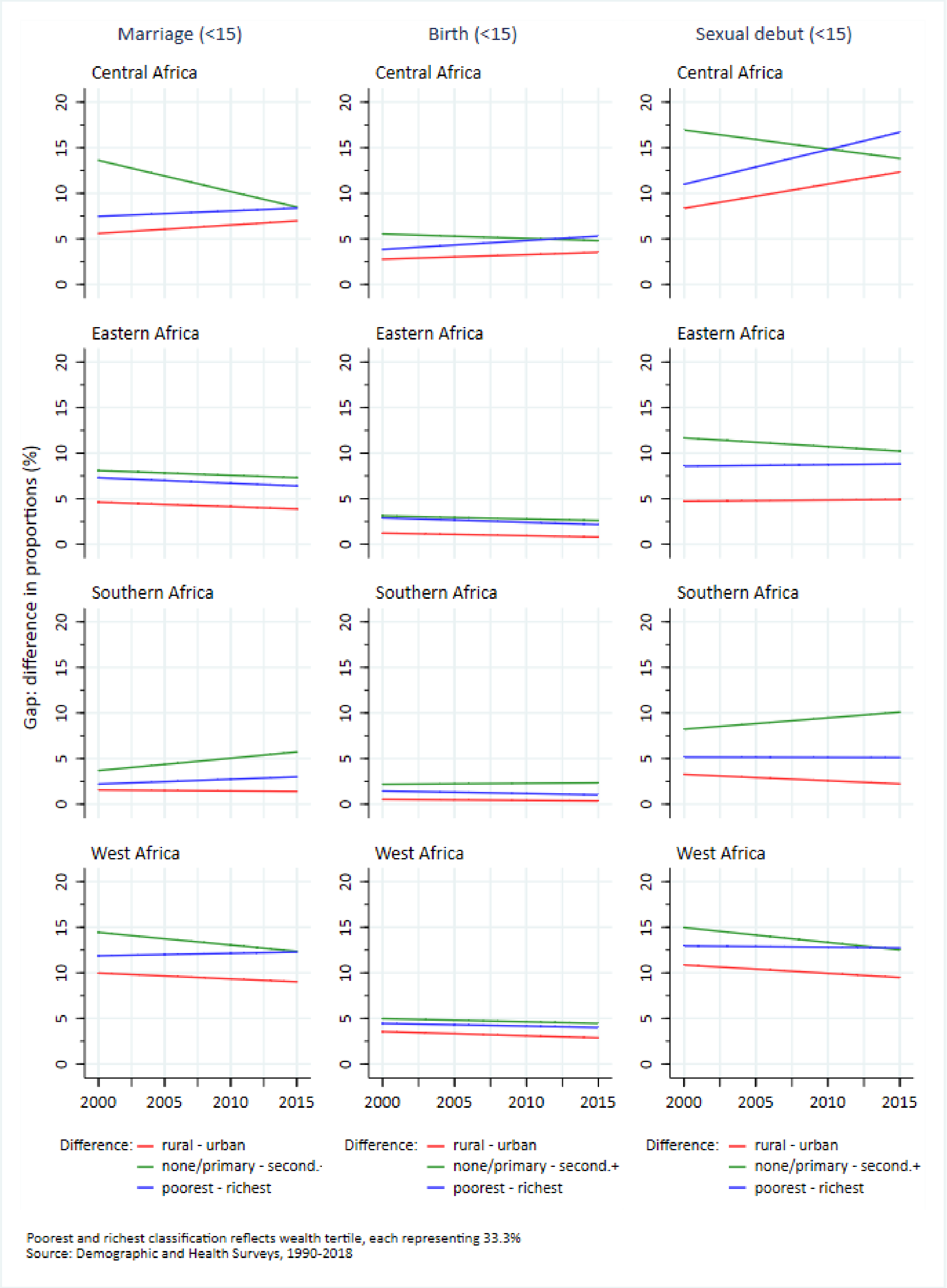

Figure S8. Child marriage among adolescent girls (age 18): patterns of inequalities by residence and socioeconomic characteristics, most recent surveys between 2010-2018, 33 countries, sub-Saharan Africa

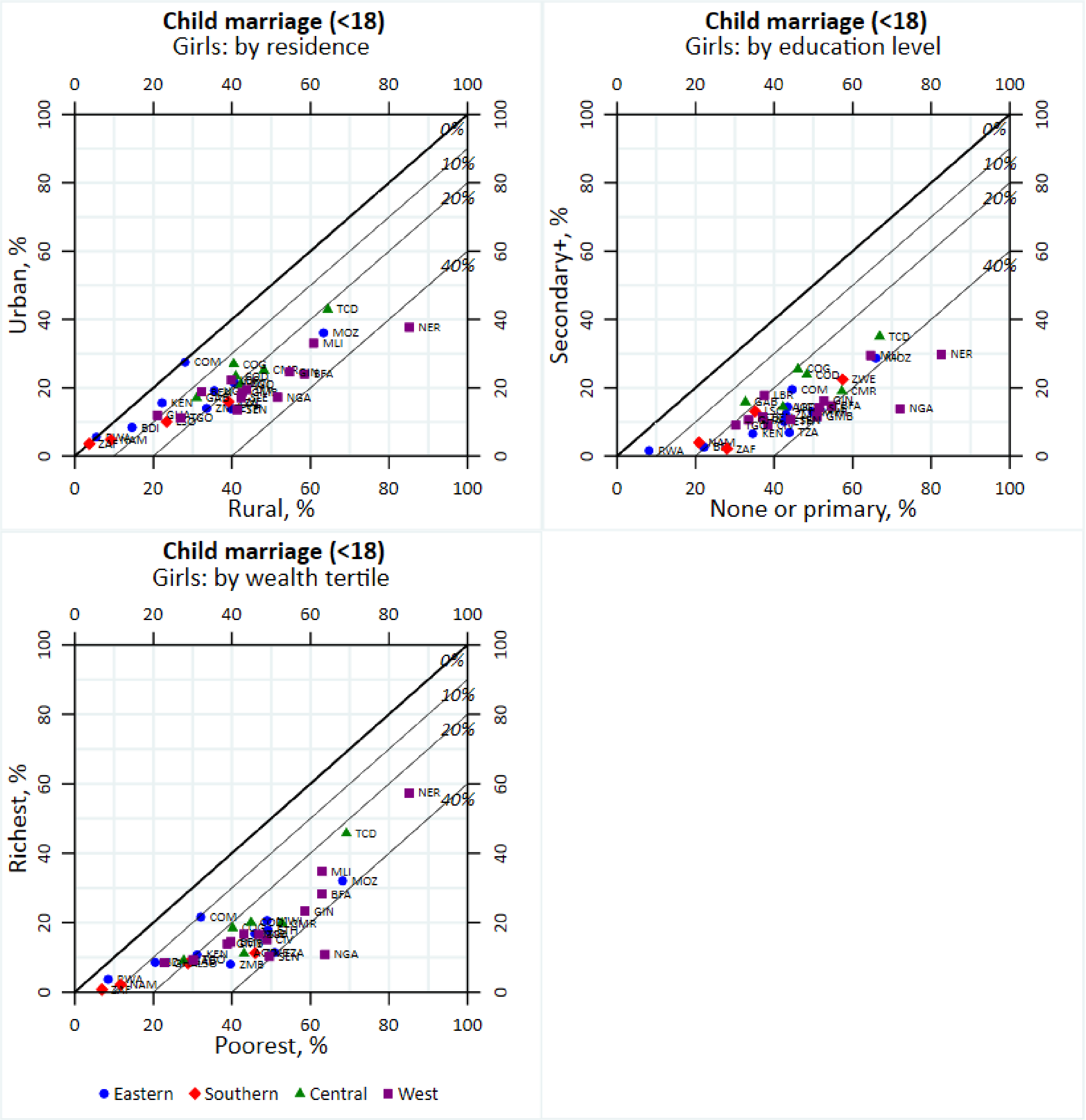

The diagonals correspond to differences in proportions of 0%, 10%, 20% and 40%.
Source: Most recent DHS surveys conducted between 2010-2018.

Figure S9. Childbearing among adolescent girls (age 20): patterns of inequalities by residence and socioeconomic characteristics, most recent surveys between 2010-2018, 33 countries, sub-Saharan Africa

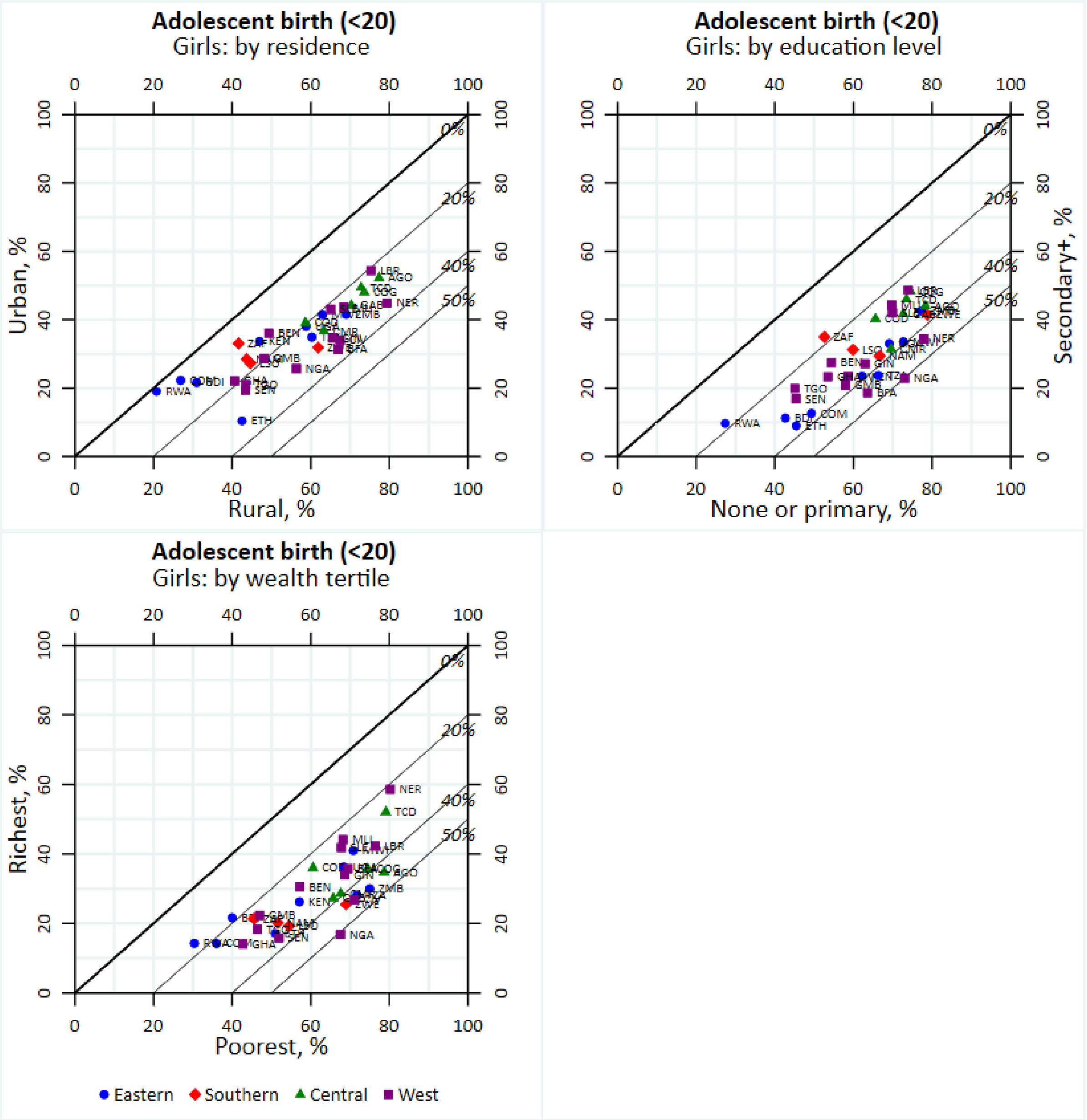

The diagonals correspond to differences in proportions of 0%, 20%, 40% and 50%.
Source: Most recent DHS surveys conducted between 2010-2018.

Figure S10. Sexual debut (age 18): patterns of inequalities by residence and socioeconomic characteristics, most recent surveys between 2010-2018, 33 countries, sub-Saharan Africa

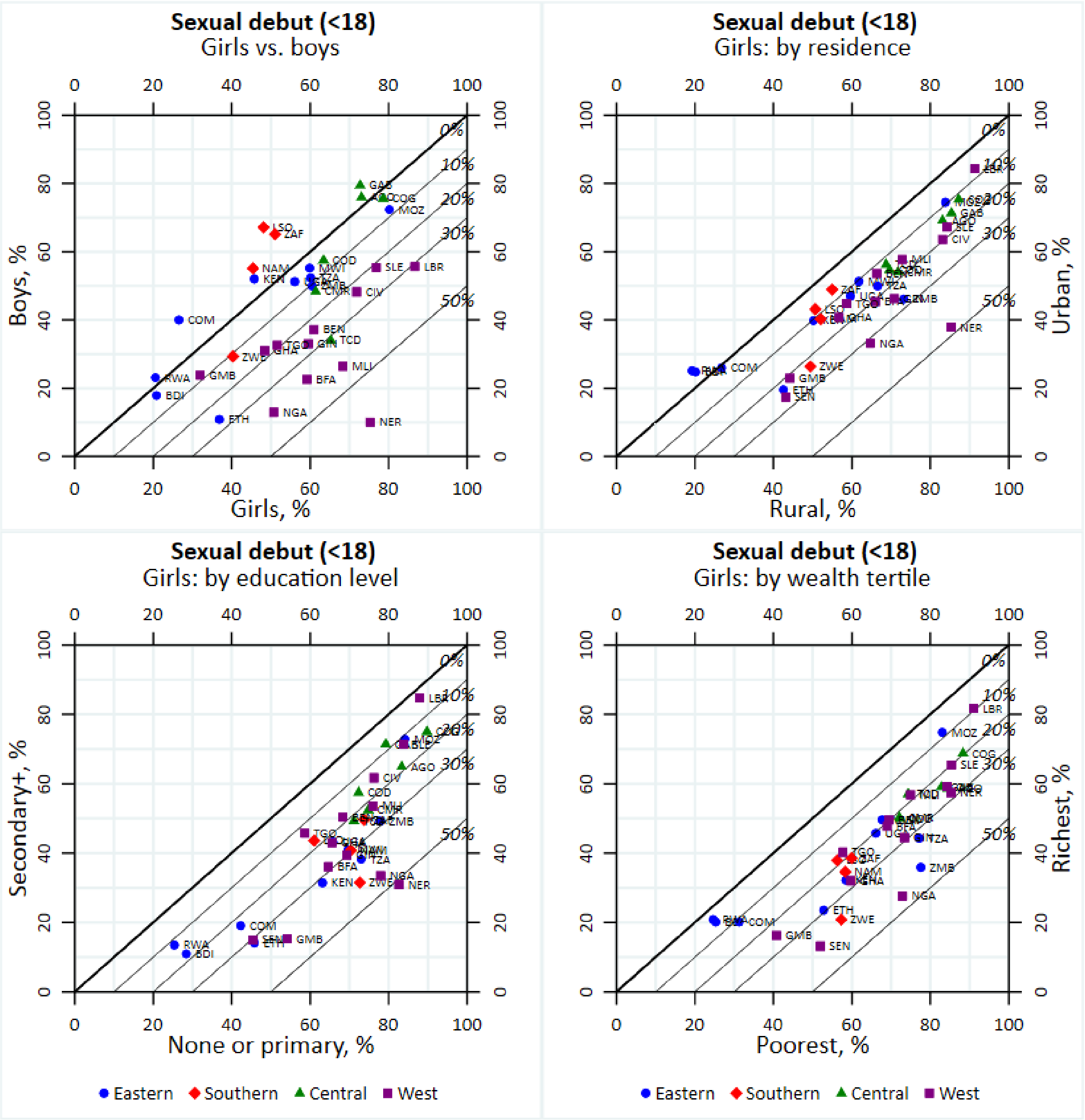

The diagonals correspond to differences in proportions of 0%, 10%, 20%, 30% and 50%.
Source: Most recent DHS surveys conducted between 2010-2018.

Figure S11. Early marriage among adolescent girls (age 15): patterns of inequalities by residence and socioeconomic characteristics, most recent surveys between 2010-2018, 33 countries, sub-Saharan Africa

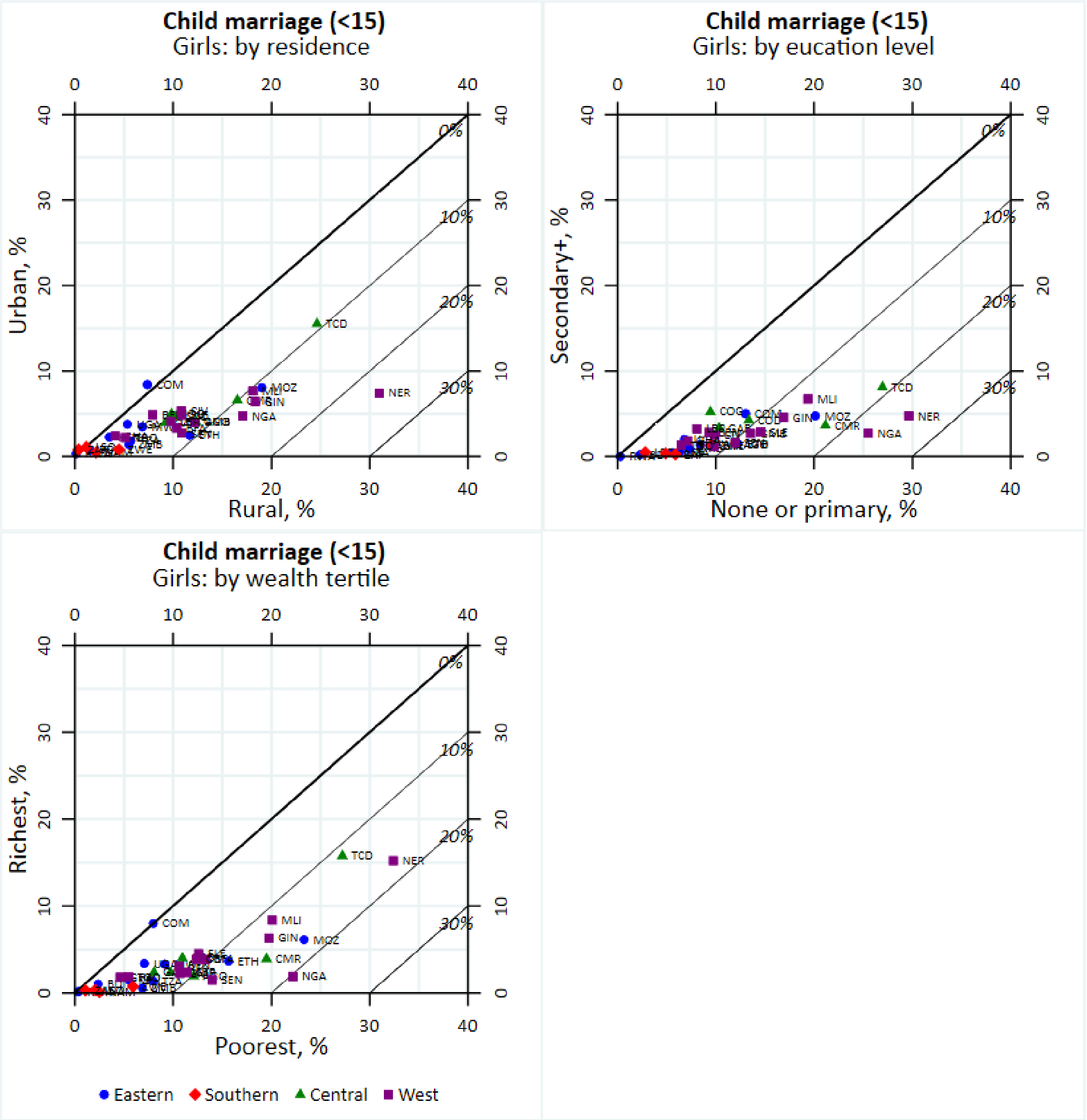

The diagonals correspond to differences in proportions of 0%, 10%, 20% and 30%.
Source: Most recent DHS surveys conducted between 2010-2018.

Figure S12. Early childbearing among adolescent girls (age 15): patterns of inequalities by residence and socioeconomic characteristics, most recent surveys between 2010-2018, 33 countries, sub-Saharan Africa

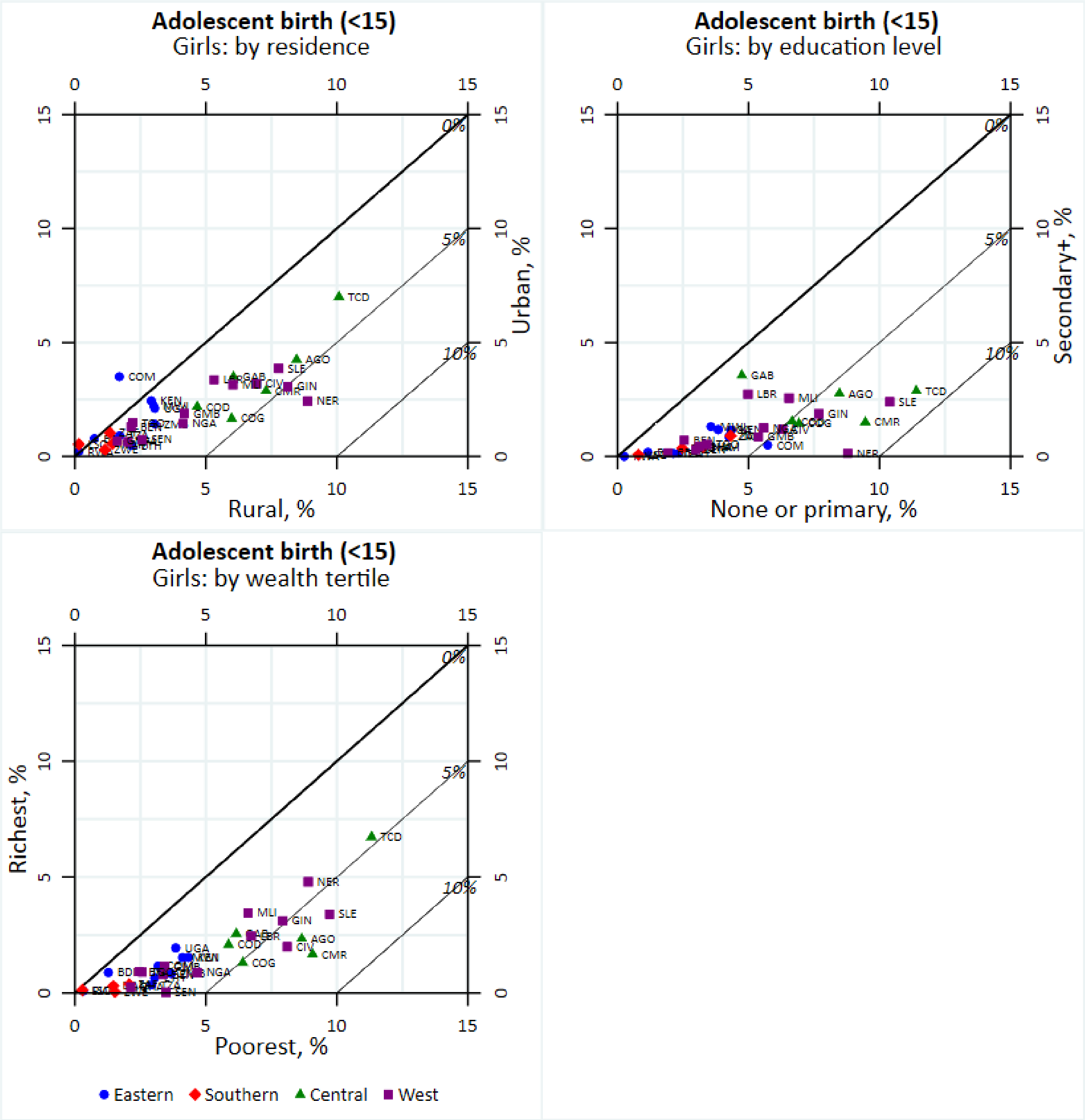

The diagonals correspond to differences in proportions of 0%, 5% and 10%.
Source: Most recent DHS surveys conducted between 2010-2018.

Figure S13. Early sexual debut (age 15): patterns of inequalities by residence and socioeconomic characteristics, most recent surveys between 2010-2018, 33 countries, sub-Saharan Africa

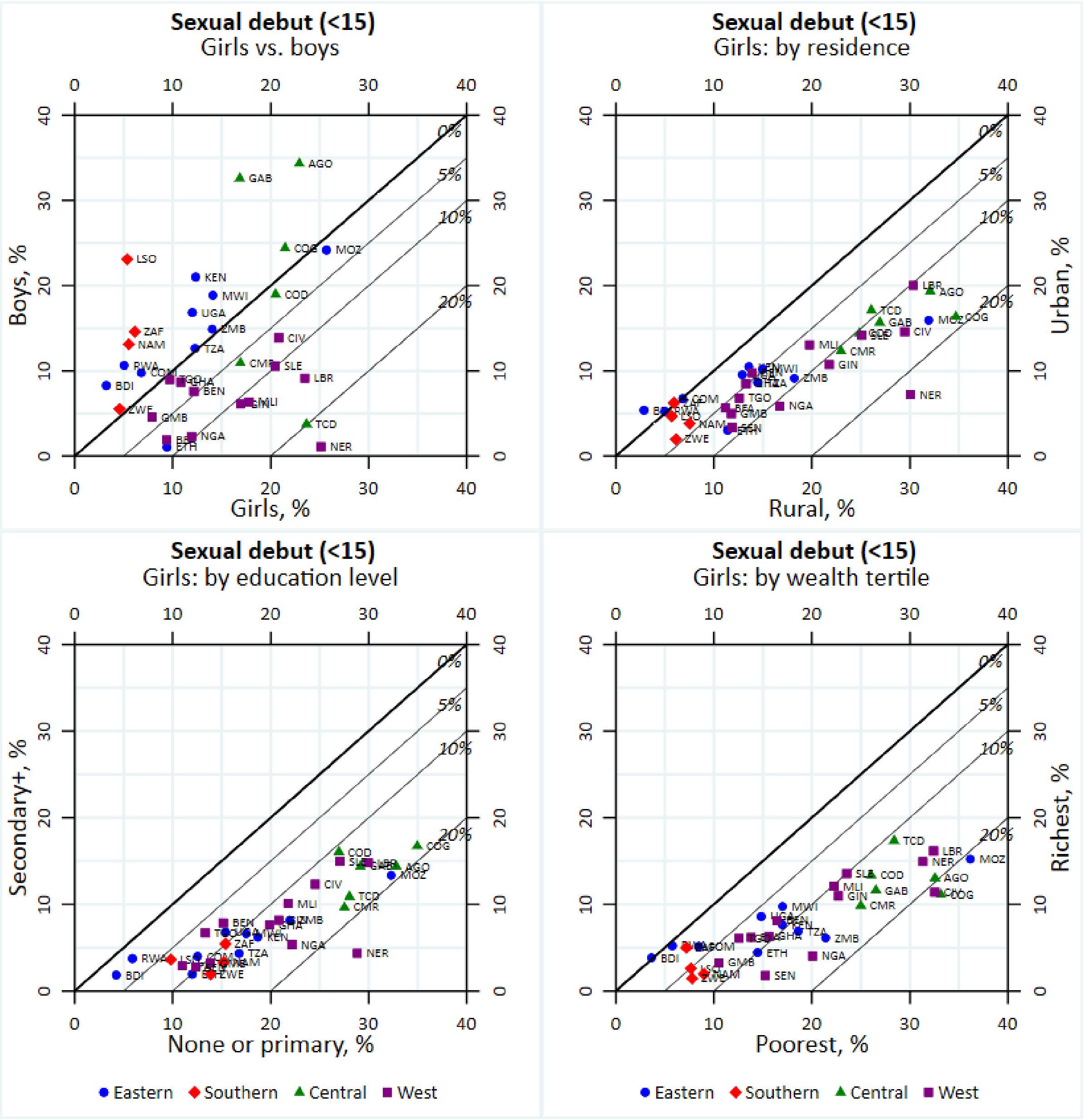

The diagonals correspond to differences in proportions of 0%, 5%, 10% and 20%.
Source: Most recent DHS surveys conducted between 2010-2018.

Figure S14. Patterns in urban-rural differences in early marriage (age 18) and childbearing (age 20) among adolescent girls, most recent survey between 2010-2018, 33 countries, sub-Saharan Africa

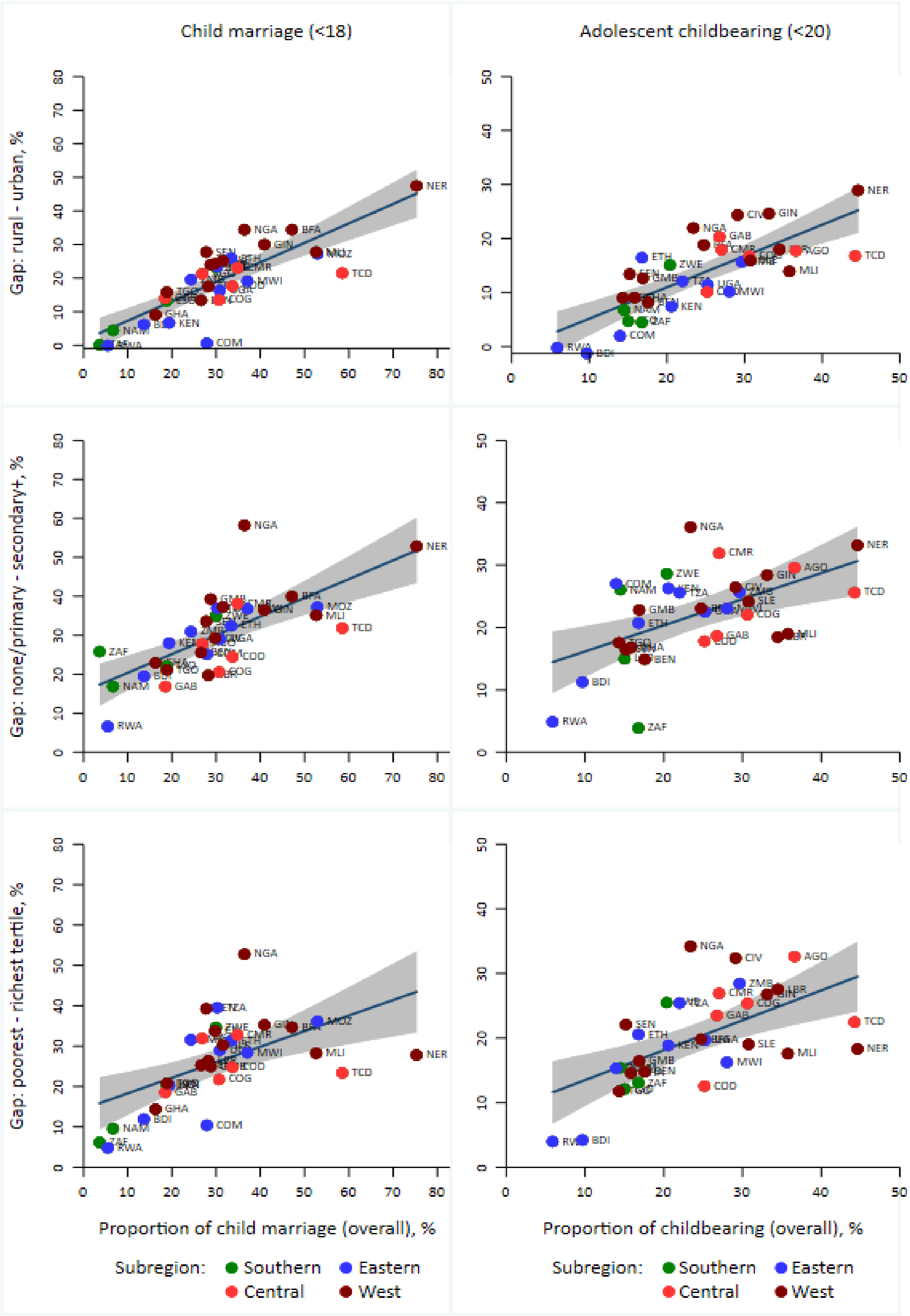

Source: Most recent DHS surveys conducted between 2010-2018.

Figure S15. Patterns in child marriage and childbearing against sexual debut in adolescent girls, most recent survey between 2010-2018, 33 countries, sub-Saharan Africa

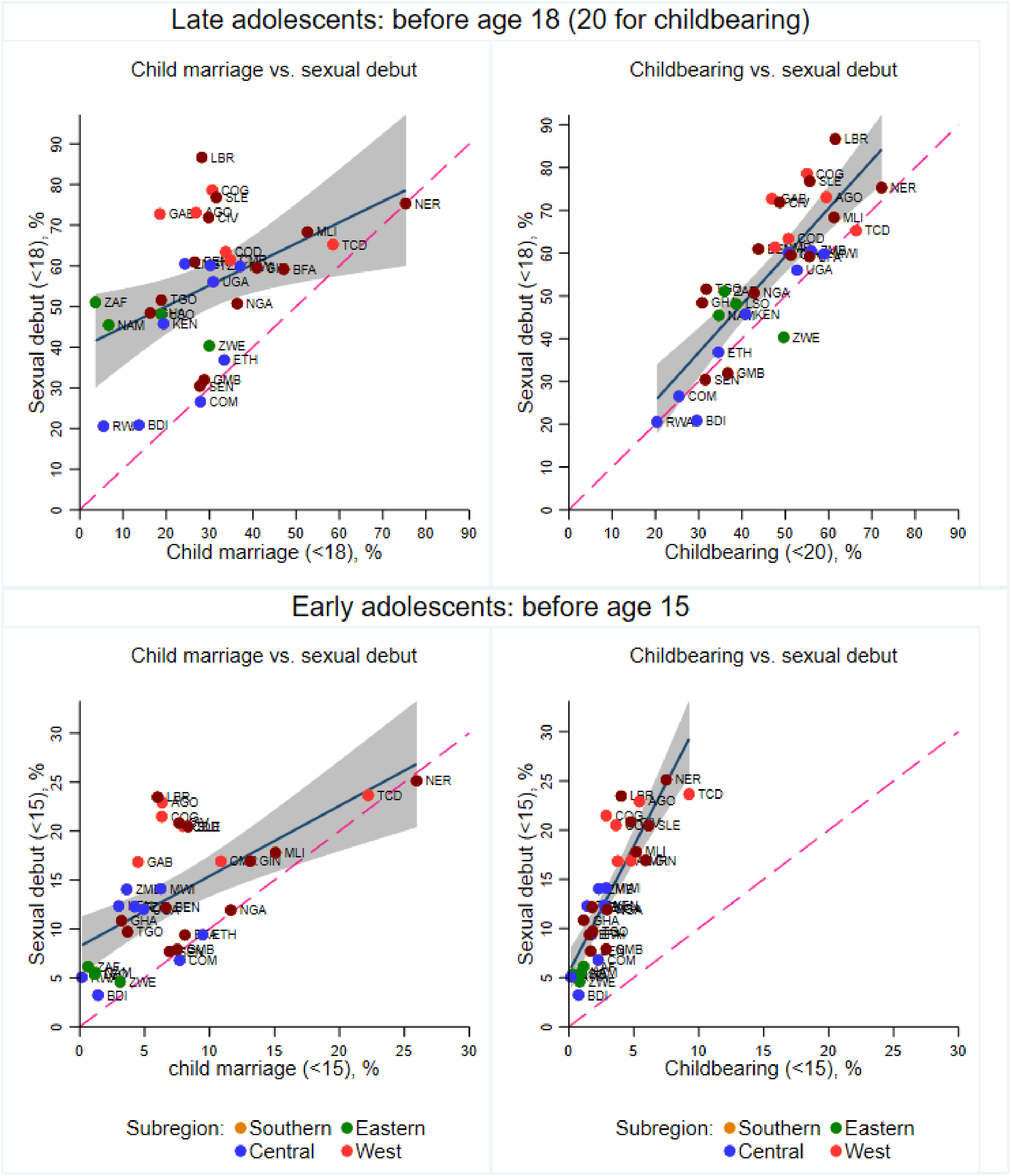

Source: Most recent DHS surveys conducted between 2010-2018.

Figure S16. Levels and trends of child marriage (age 18), childbearing (age 20), and sexual debut (age 18) for each country, obtained from survival analysis using DHS surveys data from 37 countries between 1990-2018, 37 countries, sub-Saharan Africa.

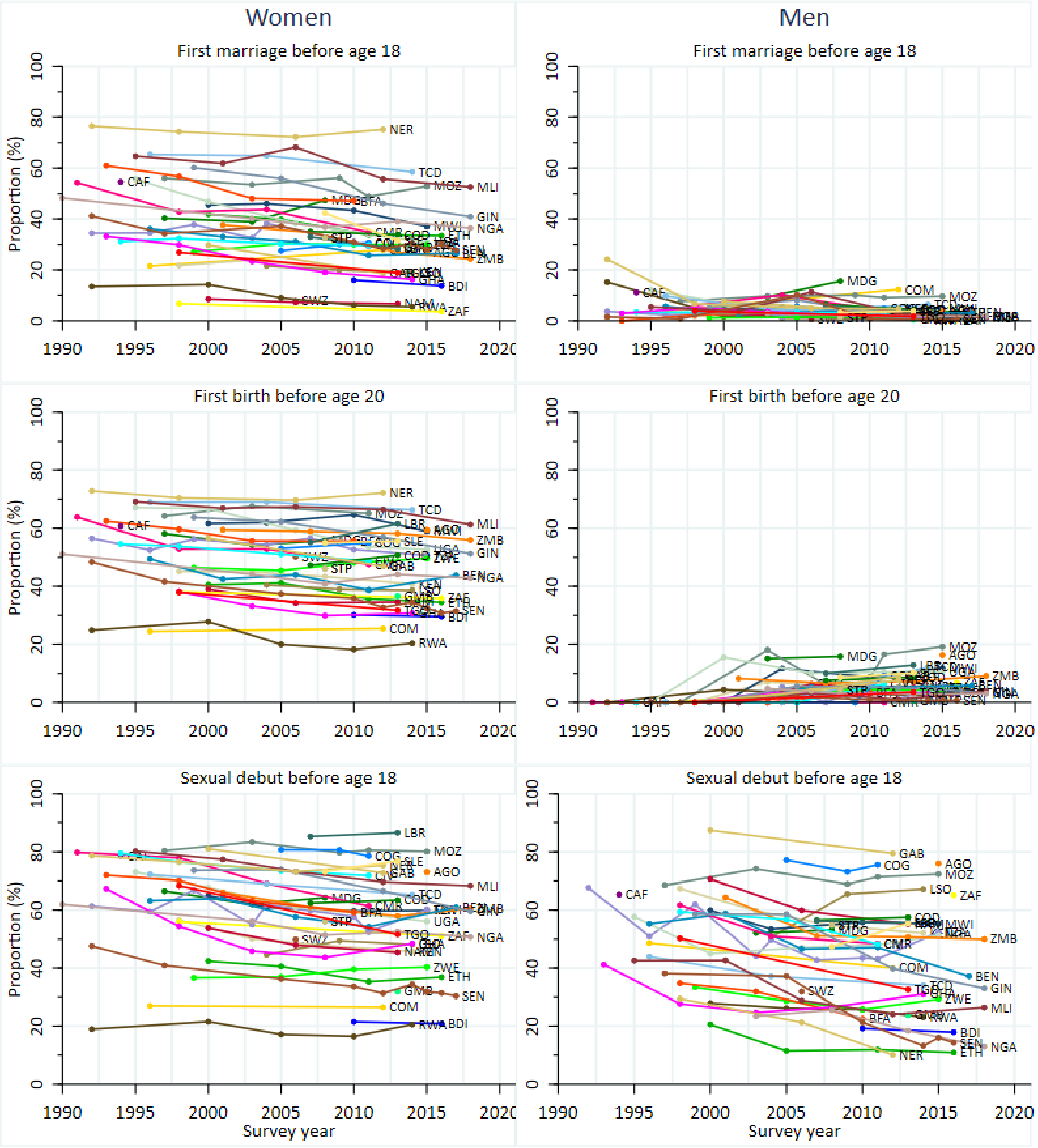

Source: Demographic and Health Surveys conducted between 1990-2018.

Figure S16. Levels and trends of early marriage, childbearing and sexual debut before age 15 for each country, obtained from survival analysis using DHS surveys data collected from 37 countries between 1990-2018, 37 countries, sub-Saharan Africa.

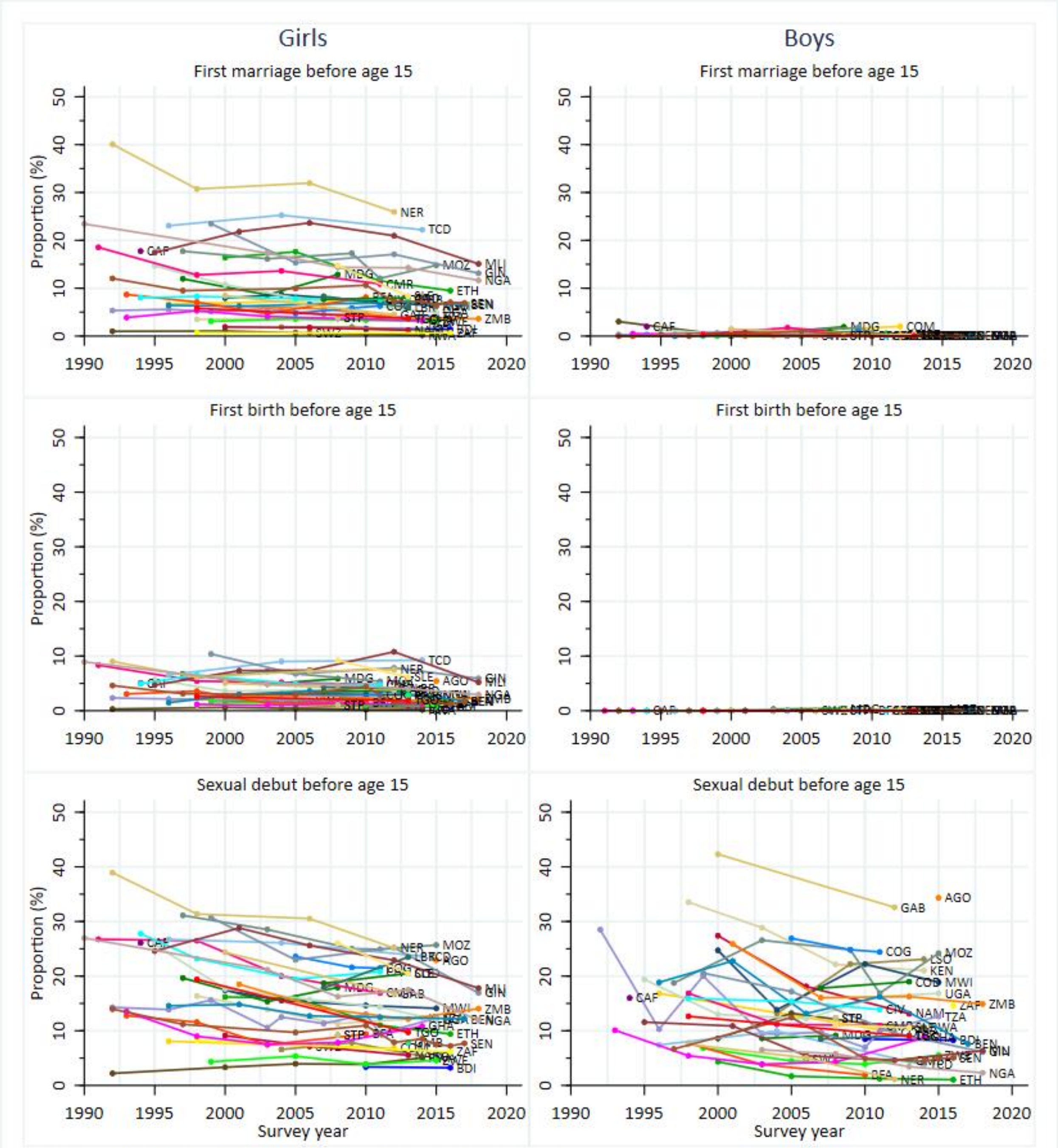

Source: Demographic and Health Surveys conducted between 1990-2018.

References

1. Manning WD, Brown SL, Payne KK. Two Decades of Stability and Change in Age at First Union Formation: Stability and Change in Age at First Union. Fam Relat. 2014;76:247–60.

2. Graubard BI, Korn EL. Modelling the sampling design in the analysis of health surveys. Stat Methods Med Res. 1996;5:263–81.

3. Gelman A, Hill J. Data analysis using regression and multilevel/hierarchical models. / Andrew Gelman, Jennifer Hill. Cambridge; New York : Cambridge University Press, 2007. New York: Cambridge University Press; 2007.

4. Greene WH. Econometric Analysis. 6th ed. Upper Saddle River, N.J.: Prentice Hall; 2008.
